# Does Distance Matter? How Physical and Social Distance Shape Our Perceived Obligations to Others

**DOI:** 10.1162/opmi_a_00138

**Published:** 2024-05-05

**Authors:** Julia Marshall, Matti Wilks

**Affiliations:** Department of Psychology and Neuroscience, Boston College, Chestnut Hill, MA, USA; Department of Psychology, University of Edinburgh, Edinburgh, UK

**Keywords:** development, social cognition, distance, obligation

## Abstract

Debates within moral philosophy have long centered on the question of whether we are more obligated to help those close to us compared to those who are farther away. Despite these debates, we have little understanding of our psychological intuitions about these issues. In the current study, we presented adults and children (5- to 9-year-olds) in the United States (*N* = 406) with hypothetical scenarios involving pairs of socially and physically close and far strangers and asked about their obligations to help one another. In general, younger children (∼6-year-olds) were more inclined to describe strangers as obligated to help one another compared to older children (∼8-year-olds) and adults. For physical distance, we documented an age-related trend where younger children were less inclined to consider physical distance when ascribing obligations to help compared to older children and adults. For social distance, we found different results depending on how social distance was manipulated. In Study 1, where social distance was manipulated via mere similarity, we found an age-related effect where adults, but not younger or older children, judged that individuals are more obligated to help socially close others relative to far ones. In Study 2, where social distance was manipulated via explicit group membership, we did not find an age trend. Instead, participants generally described individuals as more obligated to help an ingroup member relative to an outgroup one. These results demonstrate that the tendency to deny obligations towards distant others is a belief that emerges relatively late in development.

## INTRODUCTION

Imagine that you are rushing to work and come across a child drowning in a shallow pond. You can save this child, but in doing so you will ruin your new $50 shoes and risk being late for work. You are the only person around who can help. Should you wade in and save her? (Singer, 1972). Most people emphatically say yes. In fact, many would consider you a kind of moral monster if you did not. Many of us agree that we should make a small sacrifice if it can save the life of a child. However, according to the World Health Organization, more than 5 million children under the age of five died from preventable disease in 2020 alone (World Health Organization, 2022). In 2019, that number was 5.2 million (World Health Organization, 2020).

Why then do we feel differently about the child drowning in front of us compared to those dying of preventable diseases around the world? One explanation among many is *distance*; the child in the pond is immediately in front of us while children dying of preventable diseases are thought of as far away, often in another country. Does distance lessen our obligation to help? This question has been a topic of much debate in philosophy and ethics, examining whether or not physical distance should bear on our obligations towards others (Igneski, 2001; Kamm, 2007; Singer, 1972; Unger, 1996). This debate expands beyond physical distance, with scholars also questioning the role of temporal distance (i.e., those who will live in the future) (Kavka, 1982; Parfit, 1984) or evolutionary distance (i.e., non-human animals) (Singer, 1975) in shaping our obligations to others.

From a psychological perspective, we certainly appear to be affected by distance. Our responses to the plights of our friends and neighbors differ from how we react to strangers in faraway lands. Research shows that, in some cases, we will donate less money to those who are far away (i.e., physical distance; Gillis & Hagan, 1983; Kogut et al., 2018; Levine & Thompson, 2004) and that we are less generous to those we see as different from us (i.e., social distance; Everett et al., 2015; Heiphetz & Young, 2019; Stürmer et al., 2006). Other work shows that we feel less empathy and compassion to beings who are more evolutionary distinct from us (phylogenetic distance; Miralles et al., 2019). However, past research finds that our behaviors and emotions often do not always reflect our moral judgments (Godin et al., 2005; Smith et al., 2013). As such, our intuitions about our *obligations* to others appear to represent a distinct area of psychological exploration that may offer unique insights into moral and social cognition (Tomasello, 2019; Yu et al., 2019).

Although a large body of research has investigated our moral judgments more broadly (e.g., Cushman, 2008; Cushman et al., 2006) and also our obligation judgments more specifically (e.g., McManus et al., 2020, 2021), little work—at least to our knowledge—has examined how *distance* in particular shapes our perceived obligations to others. Of relevance, Baron and Miller (2000) surveyed university students from the United States and India about their moral responsibility to donate bone marrow to someone who was either a stranger from a country on the other side of the world, a stranger living in the same town, or a first cousin living in the same town. Across all conditions, students from India indicated a greater moral responsibility to donate than students from the United States, demonstrating the role of culture in shaping our judgments about obligation. However, both groups felt less obligated to help a stranger who lived on the other side of the world than a stranger who lived in the same town, suggesting that physical distance does shape adults’ judgments of obligation.

While these results demonstrate distance bias in our perceived obligations to others, this finding may also be explained by another factor: social distance. It is likely that someone who lives close to us is going to be more socially similar to us, to share our beliefs, values, and practices (Pagel & Mace, 2004). For example, people living close to one another in the northeast United States may feel more obligated to help one another—not because of physical distance per se—but because they feel a kinship toward such individuals given that they are similar to one another on many social dimensions (i.e., liking lobster rolls and the New England Patriots). Of relevance, Nagel and Waldmann (2013) revealed that physical distance does not influence prosocial helping when it is isolated from shared group membership—as well as informational directness (i.e., salience of the victim’s need) and increased efficaciousness (i.e., likelihood of helping effectively).

As such, despite long standing philosophical debate, it remains unclear how both physical and social distance, together and separately, shape our perceived obligations to others. It also remains unclear at what point in development we begin to consider physical and social distance in our obligation judgments. Past work has found cultural differences in individuals’ perceived obligation judgments to help others (e.g., Baron & Miller, 2000). These differences raise questions about our initial state: how do we reason about obligations on the basis of distance early in life when we have been exposed to relatively less social input that could shape our beliefs? On the one hand, we may be initially inclined to consider distance when making judgments about helping behavior and later reject it as a relevant factor for determining obligations (Wynn et al., 2018). This possibility would suggest that distance is a foundational facet of social judgment that is later moderated through either social or cognitive mechanisms. Alternatively, we may initially disregard distance and learn to care about it through social or cognitive mechanisms (as is the case for other kinds of prosociality; e.g., Blake et al., 2016). This would suggest that social learning and maturation play a role in encouraging children to consider distance when they would otherwise not. Addressing these possibilities requires examining whether, and to what degree, children consider distance in their judgments of obligations.

Of course, we are not the first to consider questions regarding how children think about social and physical distance. With respect to social distance, it is clear that infants and children are capable of distinguishing between different social groups and relationships, although much of this work does not address obligation specifically. In particular, preverbal infants infer that individuals who speak the same language (Liberman et al., 2017) or share saliva (eating, kissing) will affiliate with one another (Thomas et al., 2022). Moreover, infants appear to understand that group membership matters for making predictions about social behavior. For example, preverbal infants expect those who are visually depicted as members of the same group to act similarly to one another (Powell & Spelke, 2013), and 17-month-olds expect those who share a novel identity (“a bem”) to help one another more than those who do not (Jin & Baillargeon, 2017).

Building on this infant work, children infer that a character who exhibits knowledge about culturally-specific practices (such as a game) but not general knowledge (such as the sky being blue) are likely to share group membership (i.e., live in the same place or speak the same language; Soley & Köseler, 2021). Moreover, children also appear to use group membership to shape their judgments about social expectations (Chalik & Rhodes, 2020). For example, 5-year-olds think that those who share social group membership (such as being religious or not) are more likely to share psychological properties (such as liking to play “zigo”) (Diesendruck & HaLevi, 2006). Even in the absence of explicit group labels, children between the ages of 3 and 9 also think that those who merely share preferences (whether people eat the same food or not) will be more likely to be friends and share other unrelated preferences, like flying kites, than those who do not (Jordan & Dunham, 2021). Collectively, this work suggests that children make rich social inferences about individuals on the basis of both group membership and similarity.

To our knowledge, there is far less research regarding whether infants and children understand physical distance as a concept. Liu et al. (2017) found that infants inferred whether an agent preferred a certain object depending on whether that agent cleared higher barriers, climbed steeper ramps, or jumped wider gaps to reach that object. These data suggest that infants understand that traversing longer paths reflects greater effort on behalf of an agent. Together, these studies suggest that, from a very young age, children draw on social (e.g., explicit group membership) and non-social (e.g., distance) information to make inferences about how agents engage with the world.

While this research is fundamental in helping us understand how infants and children think about social expectations, they do not explore obligation specifically. It is possible that infants and children grasp certain concepts, such as physical and social distance, but that such an understanding minimally affects children’s broader sense of social obligation. Findings about social obligations are more mixed. For example, some work finds that younger children consider social relationships when making predictions about the likelihood of helping but not when making judgments about the obligatory nature of helping (Marshall et al., 2020b). Further, children also evaluate equal resource distribution amongst groups as nicer than biased distribution but, with age, become more likely to expect agents distribute resources unequally (DeJesus et al., 2014). By contrast, other studies find that both younger and older children consider ingroup members more obligated to help one another compared to outgroup members when group membership is manipulated through minimal (Chalik & Rhodes, 2020) and real-world groups, such as race and gender (Weller & Lagattuta, 2013, 2014; but see Hachey & Conry-Murray, 2020 for contrary results).

Collectively, the reviewed research regarding children’s reasoning about social obligations paints a mixed picture. Some work shows that infants and children appear to understand that social context (such as whether two individuals are a part of the same group) relates to helping behavior (Jin & Baillargeon, 2017). Adults also judge that individuals have a greater obligation to help close others relative to distant ones (e.g., Baron & Miller, 2000). This work points toward the possibility that children, regardless of age, may differentiate between someone’s obligation to help a closer individual relative to a further one. Yet, some work demonstrates that, despite young children’s understanding of social groups, they are far less inclined to consider certain such factors (e.g., social relationships) when determining obligations specifically. One possible explanation of this finding is that younger children are more inclined to apply a general heuristic that helping is obligatory when ascribing obligations to help compared to older children who may express greater nuance (Dahl et al., 2020; Marshall et al., 2020b). If a similar pattern were to hold with respect to social distance, we would expect to find that older children and adults, but not younger children, will prioritize close over distant others. We thus consider a direct investigation critical to understanding how physical and social distance independently, as well as the interaction between the two, shape children’s and adults’ perceptions of obligations to others.

### The Current Research

Here we aim to investigate the role of distance in shaping our perceived obligations to others. In particular, we explore how children between the ages of 5 and 9 and adults in the United States think about our obligations to others as a function of their physical and social distance. By *physical distance*, we mean the literal distance between two individuals. By *social distance*, we mean the social similarity between two individuals on one or many dimensions, including (but not limited to) social group membership, behaviors, preferences, customs, and values. By examining both physical and social distance, we aim to better understand the developmental origins of our perceived obligations to others and, further, shed light on the interplay between discussions in ethics and psychological intuitions of the general public.

## STUDY 1

In Study 1, we aimed to provide insight into the emergence of children’s and adults’ intuitions about obligations to help close and distant others—a topic that has deep philosophical relevance but has received little psychological examination. In line with prior work with adults (Baron & Miller, 2000), we anticipated that both children and adults would generally take physical and social distance into account when ascribing obligations. As past work suggests that younger children are less likely to take social relationships into account when considering obligations to others (Dahl et al., 2020; Marshall et al., 2020a, 2020b; Miller et al., 1990; but see Marshall et al., 2022 for cultural variation), we considered that younger children may be less discerning *in general*. As such, we predicted that older children would be more likely to consider physical and social distance when ascribing obligations relative to younger children.

### Method

#### Participants.

As outlined in our pre-registration (https://osf.io/muyq6), we collected data from a sample of children and a comparative sample of adults.

##### Children.

We planned to test approximately 20 children per categorical age who passed comprehension checks, resulting in a planned sample of 100 children aged 5–9 years. We stopped collecting data on the day in which the last child was tested using this criterion, which resulted in a final sample of 108 children (60 females, 48 males; *M*_age_ = 7.54, *SD*_age_ = 1.45). All child participants were tested online over Zoom in the Summer of 2020. The majority of the sample comprised White individuals (*n* = 72). The remaining identified as Asian (*n* = 6), Black (*n* = 7), Hispanic (*n* = 4), other (*n* = 7), or their racial-ethnic background was unknown (*n* = 12). We tested an additional 14 children but excluded them either because they failed comprehension checks (*n* = 5, which are explained below), were too old or too young (*n* = 7), or because we did not receive proper consent (*n* = 2). A sensitivity analysis revealed that our final sample of 108 had 95% power to detect a small to medium effect size (Cohen’s *d*_z_ = .35) of either Physical (or Social) Distance and 95% power (alpha = .05) to detect a small to medium-sized (Cohen’s *f* = .18) interaction between Age Group and Distance (i.e., Physical Distance × Age Group, Social Distance × Age Group).

##### Adults.

We tested a total of 101 adult participants but excluded 5 because of comprehension check failures, resulting in a total sample of 96 adult participants. Fifty participants identified as female, 42 identified as male, 4 indicated other; *M*_age_ = 29.15, *SD*_age_ = 9.87. The majority of the sample comprised individuals who were White (*n* = 48). The remaining identified as Asian (*n* = 22), Black (*n* = 6), Hispanic (*n* = 12), mixed racial-ethnic backgrounds (*n* = 7), or indicated other (*n* = 1).

#### Procedure and Materials.

All child participants were tested on Zoom in the presence of a live experimenter. The full verbatim study materials are available on Open Science Framework (OSF): https://osf.io/dztk2/.

The experimenter first introduced himself or herself and explained the process of Zoom studies, which included explaining that participants (not parents and/or siblings) should answer all the questions. Next, the experimenter instructed the participant that the study involves learning about several pairs of people. The experimenter first explained how all the pairs portrayed in the study do not know each other. This feature of the design was emphasized because we wanted to be sure that any effects of our manipulations were not due to children inferring that certain individuals (i.e., socially or physically close individuals) know one another, whereas other individuals (i.e., socially or physically far individuals) do not.

Following this, the experimenter explained the Physical Distance manipulation and the Social Distance manipulation independently (order counter-balanced across participants).

**Figure 1.**
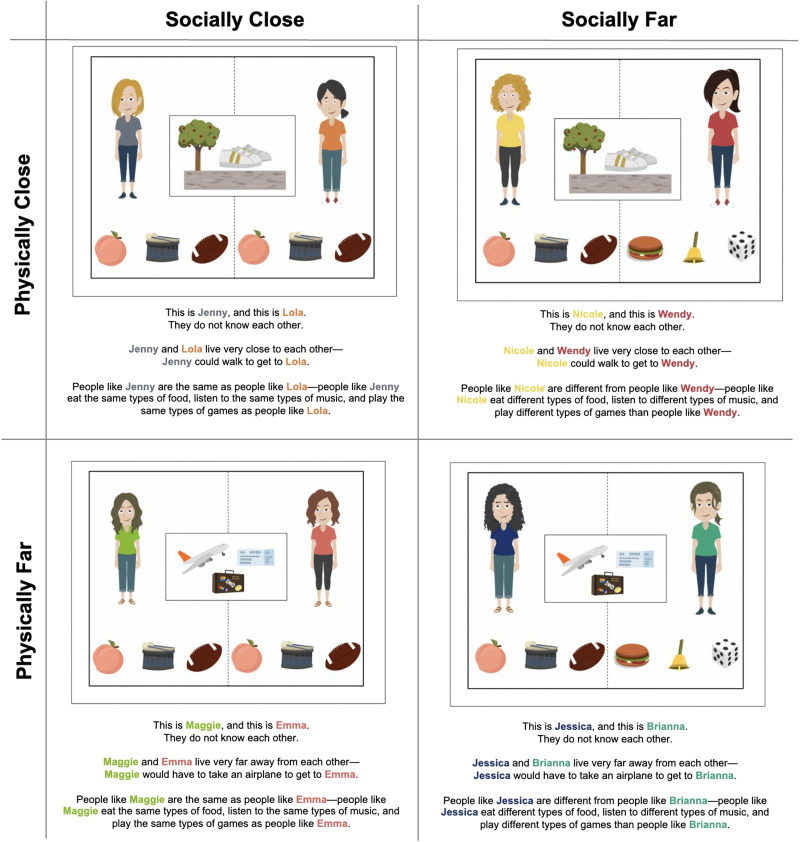
Examples of Stimuli for Study 1. *Note*. © 2019 GoAnimate, Inc.^1^

For the *Physical Distance manipulation* (close, far), the experimenter explained how some people live close to each other and some people live far away from each other. Then, in counter-balanced order, the experimenter demonstrated how some people are physically close or far from one another. For the physically close explanation, the experimenter said, “Some of the people I’ll show you live close to each other—they could walk to get to one another. See, there’s a picture of shoes, a sidewalk, and a tree” alongside an image of two individuals with a picture of shoes, a sidewalk, and a tree in between them (see Figure 1 for example). For the physically far explanation, the experimenter said, “Some people I’ll show you live far away from each other—they would have to take an airplane to get to one another. See, there’s a picture of an airplane, a bag, and a ticket” alongside an image of two individuals with a picture of an airplane, a suitcase, and a ticket in between them.

For the *Social Distance manipulation* (close, far), the experimenter explained how some “types” of people are the same or different based on the group’s social behaviors, such as the games they play, the food they eat, and the music they enjoy. For the socially close explanation, the experimenter said, “Some of the people I’ll show you are the same types of people and do things the same. They eat the same types of food, listen to the same types of music, and play the same types of games. See, these people are the same types of people—they have the same kind of food, music, and games” alongside an image of two individuals with a peach, drum, and football underneath each of them (Figure 1). For the socially far explanation, the experimenter said, “Some of the people I’ll show you are different types of people and do things differently. They eat different types of food, listen to different types of music, and play different types of games. See, these people are different types of people—they have different kinds of food, music, and games” alongside an image of two individuals—one with a peach, drum, and football underneath and one with a hamburger, bell, and die underneath.

The conceptualization of the Physical Distance manipulation (defined above) was straightforward, as physical distance was defined above as the literal distance between two individuals, and the manipulation correspondingly describes individuals as either living near or far. The conceptualization of the Social Distance (defined above) manipulation was more complex, as there are numerous ways in which individuals may be socially distant from one another. We opted to manipulate the extent to which two individuals engage in similar group-defining behaviors (music, games, foods) that have been linked to cultural practices (Soley & Köseler, 2021).

Following these explanations, the experimenter explained that helping could occur even in physically distant circumstances. Specifically, the experimenter said, “In our activity today, we are going to ask about helping. There are lots of different ways to help people. One way to help people is to donate money to those who need it. That means you can help people close to you and people far from you.” We included this component to minimize the effects of logistical challenges in shaping judgments (i.e., if it is far easier to help someone close by) as previous research has found effort modulates children’s obligation judgments (Dahl et al., 2020). However, we note that, while participants may think it *possible* to help both close and far individuals, they may still think it is *easier* to help someone close by.

Participants then responded to comprehension checks about each of these manipulations (see OSF for exact wording). In line with our preregistration, if the child responded incorrectly initially, we corrected them and asked again. If they persisted in answering incorrectly, they were excluded (*n* = 5).

The experimenter then proceeded with the main test trials, which included four scenarios^2^ followed by an obligation question (“If X needed help and Y could help her, do you think Y would have to help X? Yes or no?”). We relied on the language of “have to” to denote obligations based on previous research finding that obligation judgments are distinct from expectation and preference judgments (Kalish & Lawson, 2008; Mammen et al., 2021; Marshall et al., 2022). We also intentionally left the nature of helping unspecified (i.e., whether the person needed help because they were hungry or hurt) because we surmised that, by leaving the scenarios generic, children’s intuitions about specific types of helping will not interfere with their judgments. The four scenarios were generated from the following design: 2 (Physical Distance: close, far) × 2 (Social Distance: close, far) (Figure 1). Participants either saw scenarios that featured all males or females. These were randomly assigned and not matched to participant gender. For each pair, we counterbalanced across participants which character was on the left versus right to ensure any documented effects were not a result of the particular aesthetic of the individual.

All adult participants were recruited and paid via Prolific and completed the survey online via Qualtrics. To be eligible, participants were required to be over 18 years of age and living in the United States. The materials were identical to the materials presented to children, however participants read and responded to the stimuli themselves, rather than having an experimenter guide them.

### Results

#### Analytic Strategy.

For all analyses, we fit Bayesian logistic mixed-effects models using the brms package in R (Bürkner, 2017). This analytical strategy marks a departure from our pre-registration. We had originally specified using analysis of variance (ANOVA) tests to examine our data. Because such approaches do not accommodate binary outcomes, we pivoted to using generalized linear mixed effects (GLMMs) models rather than ANOVAs, but ran into issues doing so. In particular, the sparsity of the data in some cells of the three-way design (Physical Distance × Social Distance × Age Group) made estimating the likelihood using approximation methods impossible. This is a common issue when using maximum likelihood-based mixed-effects models, so we employed an alternative technique that is better able to deal with data sparsity, namely, Bayesian mixed-effects modeling. Bayesian statistical models allow for the incorporation of a prior distribution, which guides the estimation process without predetermining the outcome of the analysis. We specified weakly informative priors which weakly bias the model estimates towards 0 and against large effects—large effects are atypical in psychology studies (Cafri et al., 2010). Throughout both Studies 1 and 2, we conducted prior sensitivity analyses to ensure that our posterior estimates were not dictated by the particular priors we specified; in all cases, the results did not meaningfully change when doing so. Given the challenges in fitting a model to this data, we also performed posterior predictive checks for all models to ensure that the model estimates reflected the underlying data, and we also checked for chain convergence by making sure that $\hat{R}$ < 1.1. Both the prior sensitivity analyses and posterior predictive checks are located in the RMarkdown file on the OSF.

We specified a binary distribution (obligation judgments: yes = 1, no = 0) with participant ID included as a random intercept to account for individual variability across participants and model the correlation within a participant’s responses. We focused on three predictor variables: Physical Distance (close, far; within-subjects; reference group = physically close), Social Distance (close, far; within-subjects; reference group = socially close), and Age Group (younger children, older children, adults; between-subjects; reference group = younger children). All full model outputs are provided in the RMarkdown file on OSF.

The decision to look at Age Group (rather than Age, continuous) also marks a deviation from the pre-registration. Instead of examining age as a continuous variable, we included age as a categorical variable with three levels (younger children, older children, adults). Younger versus older children was determined via a median split, with younger children (*n* = 54) being between 5.00 and 7.69 and older children (*n* = 54) being between 7.70 and 9.99. We opted for this change because we were interested in the extent to which children, relative to adults, care about both physical and social distance independently and simultaneously—and the best way of addressing this question is by comparing children to adults rather than examining children separately from adults. Nonetheless, we report the planned analyses where we treat age continuously in full in the Supplemental Online Materials (SOM) and, critically, the overall pattern of results does not meaningfully change.

#### Obligation Judgments.

First, we fit a Bayesian logistic mixed-effects model that included a three-way interaction amongst the three predictors of interest—Physical Distance, Social Distance, and Age Group—and participant ID as a random effect predicting participants’ obligation judgments. In doing so, the coefficients associated with a three-way interaction were not credibly different from zero, suggesting that the age-related patterns with respect to both physical and social distance did not differ from one another; see RMarkdown for full model output.

**Figure 2.**
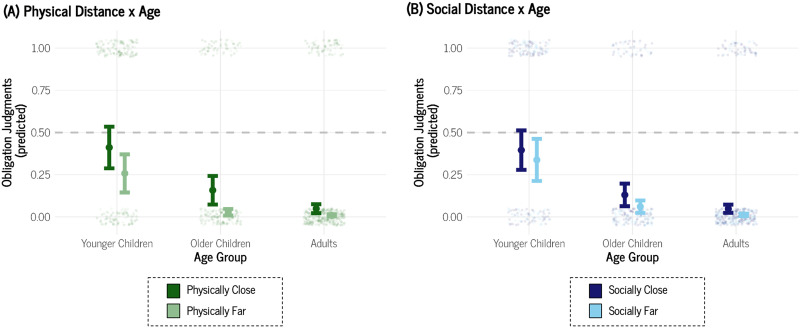
Conditional Effects Plots of (A) Physical Distance × Age Group and (B) Social Distance × Age Group for Study 1. *Note*. The dot represents the mean of the posterior distribution of the estimate. Error bars are ± 1 *SE* of the mean. The data points are jittered for readability.

Next, we fit two additional models: one included the two-way interaction between Physical Distance × Age Group and the other examined Social Distance × Age Group.^3^ The model with the Physical Distance × Age Group interaction (Figure 2A) indicated that young children did not differentiate between a bystander’s obligation to help someone physically close ($\hat{M}$ = .41, *SE* = .12) compared to someone physically far ($\hat{M}$ = .26, *SE* = .12), Odds Ratio = 0.49, 95% Credible Interval [0.23, 1.02]^4^. Interestingly, older children were more differentiating on the basis of physical distance compared to younger children, Odds Ratio = 0.31, 95% Credible Interval [0.10, 0.92]: older children considered a physically close bystander more obligated to help ($\hat{M}$ = .16, *SE* = .08) than a physically distant one ($\hat{M}$ = .03, *SE* = .02). Adults too were more inclined to consider physical distance when ascribing obligations to help compared to younger children, Odds Ratio = 0.35, 95% Credible Interval [0.13, 0.98]: adults considered a physically close bystander more obligated to help ($\hat{M}$ = .05, *SE* = .03) than a physically distant one ($\hat{M}$ = .009, *SE* = .006). Older children were not credibly more distinguishing on the basis of physical distance compared to adults, Odds Ratio = 1.16, 95% Credible Interval [0.32, 4.27]. On the whole, these data indicate that individuals become more concerned with physical distance as a factor in determining obligations in middle childhood and adulthood compared to earlier childhood.

The model with the Social Distance × Age Group interaction (Figure 2B) indicated that young children did not differentiate between a bystander’s obligation to help someone socially close ($\hat{M}$ = 0.40, *SE* = .12) compared to someone socially far ($\hat{M}$ = .34, *SE* = .12), Odds Ratio = 0.78, 95% Credible Interval [0.39, 1.58]. Unlike Physical Distance, older children were not more differentiating on the basis of social distance compared to younger children, Odds Ratio = 0.55, 95% Credible Interval [0.20, 1.56]: older children did not differ in the judgments about a socially close bystander ($\hat{M}$ = .13, *SE* = .07) versus a socially distant one ($\hat{M}$ = .06, *SE* = .04). Like Physical Distance, adults were more inclined to consider social distance when ascribing obligations to help compared to younger children, Odds Ratio = 0.30, 95% Credible Interval [0.11, 0.78]: adults considered a socially close bystander more obligated to help ($\hat{M}$ = .05, *SE* = .02) than a socially distant one ($\hat{M}$ = .01, *SE* = .007). Older children were not credibly more distinguishing on the basis of physical distance compared to adults, Odds Ratio = 0.54, 95% Credible Interval [0.16, 1.78]. On the whole, these data indicate that individuals become more concerned with social distance as a factor in determining obligations in adulthood compared to childhood.

Finally, we noticed a general pattern that younger children tended to respond more highly than older children and adults, so we fit a Bayesian logistic mixed-effects models with only Age Group as a predictor, ignoring Physical and Social Distance, and participant ID as a random intercept. Aligning with Figure 2, we found that younger children ($\hat{M}$ = .37, *SE* = .11) were more inclined to judge a bystander as obligated to help relative to older children ($\hat{M}$ = .10, *SE* = .05), Odds Ratio = 0.19, 95% Credible Interval [0.06, 0.69], and to adults ($\hat{M}$ = .03, *SE* = .02), Odds Ratio = 0.06, 95% Credible Interval [0.02, 0.18]. Older children did not differ from adults, Odds Ratio = 0.30, 95% Credible Interval [0.07, 1.24]. See Supplemental Figure 1 for visualization. These findings indicate that younger children tend to think of bystanders as more obligated to help compared to older children and adults.

### Discussion

Study 1 revealed that, relative to older children and adults, younger children are more inclined to judge helping as obligatory and less sensitive to physical distance when ascribing obligations. Although older children and adults are more inclined to describe helping a physically close individual as obligatory compared to a physically distant individual, neither older children nor adults are particularly inclined to describe helping as obligatory in general. Importantly, the developmental effect we found with respect to physical distance was generally maintained for both socially close and far situations. This finding suggests that, in contrast to past work with adults (e.g., Nagel & Waldmann, 2013), the effects of physical distance are not reducible to similarity. We also found that children (both younger and older) did not discriminate on the grounds of social distance when making judgments of prosocial obligations, while adults did.

## STUDY 2

In Study 1, we did not find an effect of social distance for either younger or older children; that is, children did not distinguish prosocial obligations on the basis of social distance. This null effect stands at odds with past work which demonstrates that children determine obligations on the basis of novel and real-world social groups (e.g., Chalik & Rhodes, 2020; Weller & Lagattuta, 2013, 2014). Study 2 aimed to investigate this apparent conflict.

One possible explanation for the lack of social distance effect may relate to the specific manipulation employed. As described above, we told participants that socially close people did the same types of things, such as playing the same games, while socially distant people did these things differently from one another. This social distance manipulation is weaker than the ‘group membership’ manipulations commonly used ingroup bias research (e.g., Chalik & Rhodes, 2018, 2020; Dunham et al., 2011). Indeed, most social group membership manipulations involve richer information, notably explicitly describing the individuals as members of the same group and visually depicting them as such.

In Study 2, we opted to bolster our manipulation of social distance to include an explicit group membership manipulation. That is, rather than explaining social closeness just in terms of similar (or dissimilar) behaviors, we explicitly described the pairs as members of the same group. Specifically, we developed two novel groups—the “Prembalts” and “Strizzers”—and designated group membership by shirt color in addition to describing similar (or dissimilar) behaviors, as in Study 1. We predicted that older children would be more likely to consider social distance in the form of explicit group membership when ascribing obligations to help, given the increased salience of our manipulation. We did not anticipate younger children to do so, given the results of Study 1. Like in Study 1, we again planned to compare children’s responses to adults’.

### Method

#### Participants.

As per our pre-registration (https://osf.io/fr5b7), we collected a sample of children and a comparative sample of adults.

##### Children.

As in Study 1 and as outlined in our preregistration, we planned to test approximately 20 children per categorical age who passed comprehension checks. This resulted in planned sample of 100 children aged 5–9 years. Using this criterion, we stopped collecting data on the day in which the last child was tested. We ultimately tested 99 total children^5^ (56 females, *M*_age_ = 7.60, *SD*_age_ = 1.47). All participants were tested online over Zoom in the summer of 2021. The majority of the sample comprised White individuals (*n* = 62). The remainder identified as Asian (*n* = 18), Black (*n* = 3), biracial (*n* = 1), Hispanic (*n* = 2), other (*n* = 8), or their ethnicity was unknown (*n* = 5). We tested an additional 11 children but excluded them either because they failed comprehension checks (*n* = 6), because of experimenter error (*n* = 2) or technical error (*n* = 1), because they had previously participated in the study (*n* = 1), or because we did not receive proper consent (*n* = 1). A sensitivity analysis revealed that our final sample of 99 had 95% power to find a small to medium effect size (Cohen’s *d*_z_ = .37) of either Physical Distance (or Social Distance) and 95% power (alpha = .05) to detect a small to medium-sized (Cohen’s *f* = .18) interaction between Age Group and Distance (i.e., physical distance, social group membership).

##### Adults.

We tested a total of 100 adult participants but excluded 2 because of comprehension check failures, resulting in a total sample of 98 adult participants (48 identified as female, 48 identified as male, 2 indicated other; *M*_age_ = 38.76, *SD*_age_ = 15.80). The majority of the sample comprised individuals who were White (*n* = 57). The remaining identified as Asian (*n* = 17), Black (*n* = 9), Hispanic (*n* = 8), mixed ethnicities (*n* = 5), or indicated other (*n* = 2).

#### Procedure and Materials.

The study was almost identical to Study 1, and the full materials are available on the OSF (https://osf.io/fr5b7). The key difference was the Social Distance manipulation. Importantly, we deployed the same Social Distance manipulation but added additional explicit information about social group membership. Here we not only described similar pairs as having similar preferences (i.e., listening to the same music, playing the same games, eating the same foods) but also described the pairs as explicitly in the same group (or not). Specifically, the experimenter said, “You know how some types of people are in the same group and how some types of people are in different groups? In today’s activity, we’re going to talk to two different groups—the Prembalts and the Strizzers.” We refer to this manipulation as Social Group, rather than Social Distance, to serve as a reminder that social group membership was explicitly manipulated.

The experimenter first explained the two groups—the Prembalts and the Strizzers: “The Prembalts (Strizzers) are in the same group because they do things the same; they eat the same types of food, listen to the same types of music, and play the same types of games. The Strizzers (Prembalts) are in the same group because they do things the same; they eat the same food, listen to the same types of music, and play the same types of games.” One group was always associated with red shirts, peaches, drums, and football, whereas the other group was also associated with green shirts, hamburgers, bells, and dice. Although in the test trials we only portrayed individuals in red shirts, we counterbalanced whether the first group was called the Prembalts (or Strizzers). The experimenter also explicitly pointed out how individuals in the same group wore the same color t-shirt: “As you can see the Prembalts (Strizzers) wear Red shirts and the Strizzers (Prembalts) wear Green shirts.”

The experimenter then introduced members of the groups—an image showing a pair of ingroup members and an image showing a pair of outgroup members—in counterbalanced order. For the ingroup members, the experimenter said, “Some of the people I’ll show you are in the same group—for example, this person is one of the Prembalts (Strizzers) and this person is also one of the Prembalts (Strizzers). They wear the same-colored shirts and do things the same—they eat the same types of food, listen to the same types of music, and play the same types of games” alongside an image of two individuals in red shirts with a peach, drum, and football underneath each one. For the outgroup members, the experimenter said, “Some of the people I’ll show you today are in different groups—for example, this person is one of the Prembalts (Strizzers) and this person is one of the Strizzers (Prembalts). They wear different colored shirts and do things differently—they eat different types of food, listen to different types of music, and play different types of games” alongside an image of two individuals—one in a red shirt with a peach, drum, and football, and one in a green shirt with a hamburger, bell, and die.

**Figure 3.**
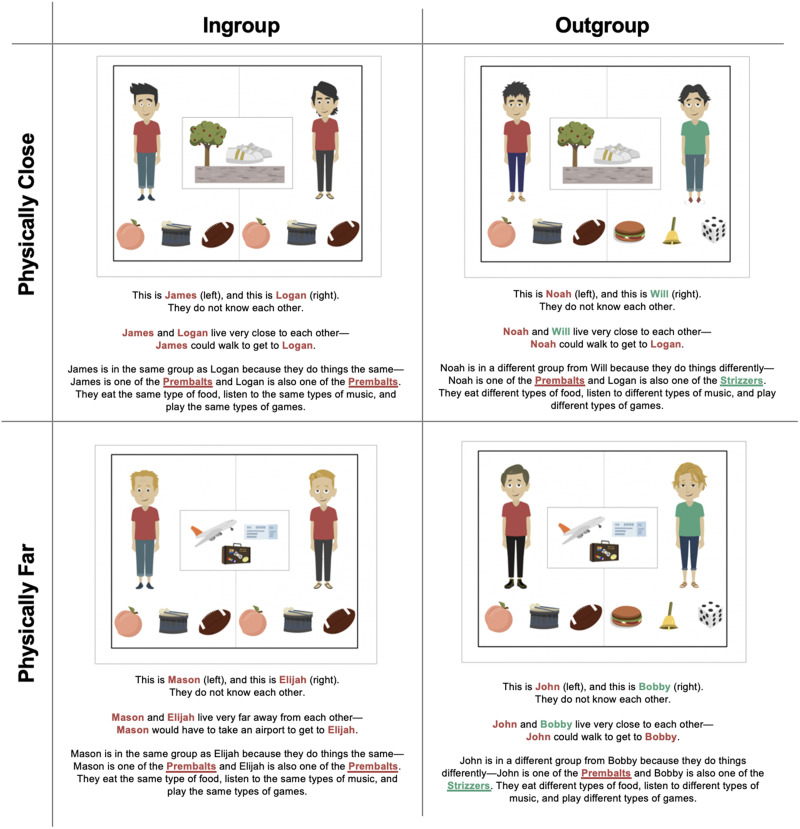
Examples of Stimuli for Study 2. *Note*. © 2019 GoAnimate, Inc.^1^

The remainder of the study was similar to Study 1 in its structure and experimental set-up. Again, participants saw four total trial scenarios (Figure 3)^6^. The only additional difference between Study 1 and Study 2 involved an additional explanation measure, which we included immediately after participants answered the questions for each story. Here we asked, “Why do you think that?” We included this item to garner exploratory insight into children’s reasoning about their decisions.

All adult participants were recruited and paid via Prolific and completed the survey online via Qualtrics. To be eligible, participants were required to be over 18 years of age and living in the United States. The materials and procedure were identical to Study 2, however participants read and responded to the stimuli themselves, rather than having an experimenter guide them.

### Results

#### Obligation Judgments.

We adopted the same analytical plan as was used in Study 1. As was the case in Study 1, we first fit a Bayesian logistic mixed-effects model that included a three-way interaction amongst the three predictors of interest—Physical Distance, Social Group Membership (previously called, Social Distance), and Age Group—and participant ID as a random effect predicting participants’ obligation judgments. In doing so, the coefficients associated with a three-way interaction were not credibly different from zero, suggesting that the age-related patterns with respect to both physical and social distance did not differ from one another; see Rmarkdown for full model output.

**Figure 4.**
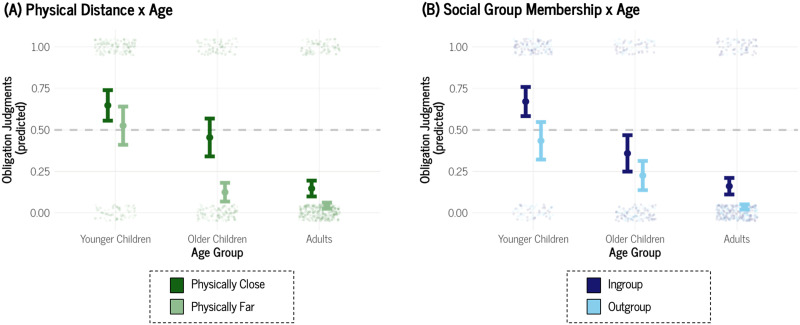
Conditional Effects Plots of (A) Physical Distance × Age Group and (B) Social Distance × Age Group for Study 2. *Note*. The dot represents the mean of the posterior distribution of the estimate. Error bars are ± 1 *SE* of the mean. The data points are jittered for readability.

Next, we fit two additional models: one included the two-way interaction between Physical Distance × Age Group and the other examined Social Group Membership × Age Group. Like Study 1, the model with the Physical Distance × Age Group interaction (Figure 4A) indicated that young children did not differentiate between a bystander’s obligation to help someone physically close ($\hat{M}$ = .65, *SE* = .09) compared to someone physically far ($\hat{M}$ = .53, *SE* = .12), Odds Ratio = 0.60, 95% Credible Interval [0.30, 1.19]. Interestingly, older children were more differentiating on the basis of physical distance compared to younger children, Odds Ratio = 0.28, 95% Credible Interval [0.10, 0.76]: older children considered a physically close bystander more obligated to help ($\hat{M}$ = .45, *SE* = .11) than a physically distant one ($\hat{M}$ = .12, *SE* = .06). Unlike Study 1, younger children and adults did not credibly differ in their willingness to consider physical distance, Odds Ratio = 0.44, 95% Credible Interval [0.17, 1.08], although adults did consider a physically close bystander more obligated to help ($\hat{M}$ = .15, *SE* = .05) than a physically distant one ($\hat{M}$ = .04, *SE* = .02). Older children were not credibly more distinguishing on the basis of physical distance compared to adults, Odds Ratio = 1.54, 95% Credible Interval [0.54, 4.35]. On the whole, these data provide a less straightforward developmental pattern. Younger children are less concerned with physical distance as a factor in determining obligations compared to older children, but the extent to which adults differentiate on the basis of physical distance is similar to both younger and older children.

The model with the Social Group Membership × Age Group interaction (Figure 4B) indicated that, unlike Study 1, young children did differentiate between a bystander’s obligation to help an ingroup member ($\hat{M}$ = .67, *SE* = .09) compared to an outgroup member ($\hat{M}$ = .43, *SE* = .11), Odds Ratio = 0.38, 95% Credible Interval [0.19, 0.75]. Younger children’s differentiation did not credibly differ from either older children’s, Odds Ratio = 1.38, 95% Credible Interval [0.54, 3.44], or adults’, Odds Ratio = 0.52, 95% Credible Interval [0.20, 1.28]. For this reason, we fit another Bayesian logistic mixed-effects model with just Social Group Membership as a main effect. In doing so, we found that participants regardless of age generally differentiated between an ingroup member ($\hat{M}$ = .32, *SE* = .06) and an outgroup member ($\hat{M}$ = .13, *SE* = .04), Odds Ratio = 0.30, 95% Credible Interval [0.19, 0.48]. On the whole, these data indicate a different pattern than in Study 1, likely as a result from our changes in the social distance manipulation. Younger children did not differentiate between socially close and far individuals in Study 1 but did so in Study 2. Older children did not consider social distance in either study, while adults considered it in both.

As was the case in Study 1, we again noticed a general pattern that younger children tended to respond more highly than older children and adults, so we fit a Bayesian logistic mixed-effects model with only Age Group as a predictor, ignoring Physical Distance and Social Group Membership, and including participant ID as a random effect. Aligning with Figure 4, we found that younger children ($\hat{M}$ = .59, *SE* = .09) were more inclined to judge a bystander as obligated to help relative to older children ($\hat{M}$ = .30, *SE* = .08), Odds Ratio = 0.29, 95% Credible Interval [0.11, 0.83], and to adults ($\hat{M}$ = .10, *SE* = .03), Odds Ratio = 0.08, 95% Credible Interval [0.03, 0.19]. Older children also differed from adults, Odds Ratio = 0.27, 95% Credible Interval [0.09, 0.73]. See Supplemental Figure 5 for visualization. These findings indicate that, generally speaking, younger children tend to think of bystanders as more obligated to help compared to older children and adults.

#### Exploratory Analyses.

Next, we wanted to examine two components of the obligation data. First, we collapsed across Study 1 and 2 to generate a more comprehensive picture of age-related changes in children’s judgments of physically close versus far individuals. We wanted to do so in part because, unlike the Social Group Membership manipulation which differed between Study 1 and 2, the physical distance manipulation was the same between the two studies. Second, we noticed that younger children did not differentiate between socially close versus far individuals in Study 1 but did so in Study 2. We wanted to assess whether this difference was statistically meaningful. Because both of these analyses are exploratory, they should be interpreted with caution.

**Figure 5.**
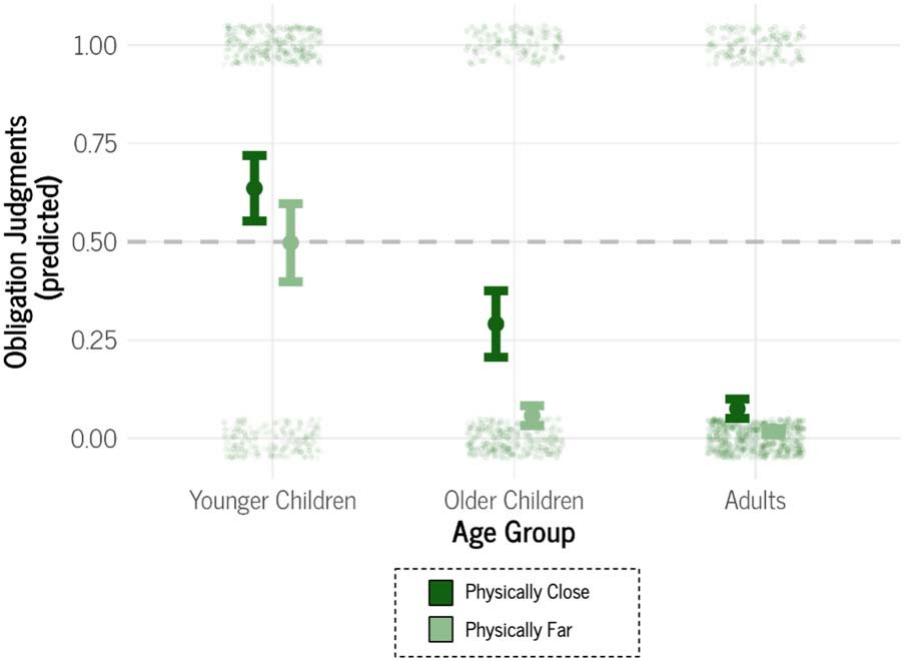
Conditional Effects Plots of Physical Distance × Age Group Collapsed Across Study 1 and 2. *Note*. The dot represents the mean of the posterior distribution of the estimate. Error bars are ± 1 *SE* of the mean. The data points are jittered for readability.

With to our first exploratory analyses, we collapsed across Study 1 and Study 2 and fit a Bayesian logistic mixed-effects model with participant ID as a random effect and included the Physical Distance (close, far; within-subjects) × Age Group (younger children, older children, and adults; between-subjects) interaction term; the outcome variable was obligation judgments. Looking at this model (Figure 5), we found consistent age-related changes. Young children (*n* = 103) differentiated between a bystander’s obligation to help someone physically close ($\hat{M}$ = .64, *SE* = .08) compared to someone physically distant ($\hat{M}$ = .50, *SE* = .10), Odds Ratio = 0.56, 95% Credible Interval [0.33, 0.97]. Importantly, older children (*n* = 104) were more differentiating on the basis of physical distance compared to younger children, Odds Ratio = 0.27, 95% Credible Interval [0.12, 0.61]: older children considered a physically close bystander more obligated to help ($\hat{M}$ = .29, *SE* = .08) than a physically distant one ($\hat{M}$ = .06, *SE* = .03). Adults (*n* = 194) too were more differentiating on the basis of physical distance compared to younger children, Odds Ratio = 0.39, 95% Credible Interval [0.19, 0.81]: adults considered a physically close bystander more obligated to help ($\hat{M}$ = .08, *SE* = .02) than a physically distant one ($\hat{M}$ = .02, *SE* = .01). Older children were not credibly more distinguishing on the basis of physical distance compared to adults, Odds Ratio = 1.46, 95% Credible Interval [0.62, 3.48]. On the whole, these data provide a more straightforward picture of the age-related effects: although younger children exhibited sensitivity to physical distance, they were far less differentiating on the basis of physical distance relative to older children and adults.

With respect to our second exploratory analyses, we fit a Bayesian logistic mixed-effect model with Social Distance (close, far; within-subjects) × Study (1, 2; between-subjects) as a predictor and with participant ID as a random effect; the outcome variable was obligation judgments. We selected for younger children because younger children were sensitive to Social Distance in Study 1 but not 2, older children were sensitive to Social Distance in neither Study 1 nor 2, and adults were sensitive in both. As a result, we wanted to address whether the change in younger children between Study 1 and 2 was statistically meaningful. The model revealed an interaction between Study and Social Distance, Odds Ratio = 0.31, 95% Credible Interval [0.11, 0.90]. In particular, younger children did not differentiate between socially close ($\hat{M}$ = .74, *SE* = .09) and far individuals ($\hat{M}$ = .66, *SE* = .12) in Study 1 when Social Distance was manipulated in an arguably more subtle manner. Younger children did differentiate between ingroup members ($\hat{M}$ = .88, *SE* = .07) and outgroup members ($\hat{M}$ = .61, *SE* = .14) in Study 2 when Social Distance was manipulated in a more explicit manner (see Supplemental Figure 9).

#### Children’s Explanations.

We also recorded children’s explanations for their obligation judgments to garner insight into why children consider certain individuals obligated or not. To make sense of this data, the two authors examined participants’ responses and generated a coding scheme (Table 1). The scheme was developed independently from participants’ responses; that is, the authors developed this scheme as way to capture children’s explanations about obligations regardless of whether the participant viewed helping as obligatory or not. The coding scheme resulted in seven explanation categories. Two independent coders blind to study hypotheses assessed all participants’ responses. Some participants’ explanations drew on multiple themes; in such cases, coders were instructed to assign the explanation a primary code depending on which theme appeared first in the explanation. Beyond the seven categorizations, coders were also instructed to code if participants indicated that they did not know or whether the coders thought the participants’ responses did not fit any of the categories. By conventional standards, there was good agreement amongst the coders, *k* = .74. Because of this, we opted to use just one coder’s assignments (chosen randomly).

**Table 1.** Participants’ Explanations as a Function of Their Obligation Judgment.

Explanation Type	Example		Frequencies	
	Yes (Obligated to help)	No (Not obligated to help)	Yes	No
Social Group	“Because even though they’re in different groups, they can still help each other”	“Because they’re in a different group”	41	25
Physical Distance	“Because even though they live far away from each other, they can still go to an airplane if Elijah is sad or hurt”	“Because they could be really far away like in England and Michigan”	35	35
Relationships	“Because even though people don’t know each other doesn’t mean you don’t have to help them”	“Because if they don’t know each other, then they might not know where their houses are”	12	23
Capacity to Help	“Because they can just video chat with each other”	“Because they live very far away, and I don’t think they can meet”	15	14
General Obligations	“Because sometimes you have to help others”	“Because you don’t have to help anybody”	14	91
Kindness	“Because it’s always nice to help people”	“Because it’s nice if you help someone but you don’t always have to do it because someone else can do it”	31	4
Emotions, Severity, Needs	“Because if he needs help and you don’t help him it would be hard for him”	“Because, if it was something not big, then he would’ve come all the way over there for nothing”	63	7

**Figure 6.**
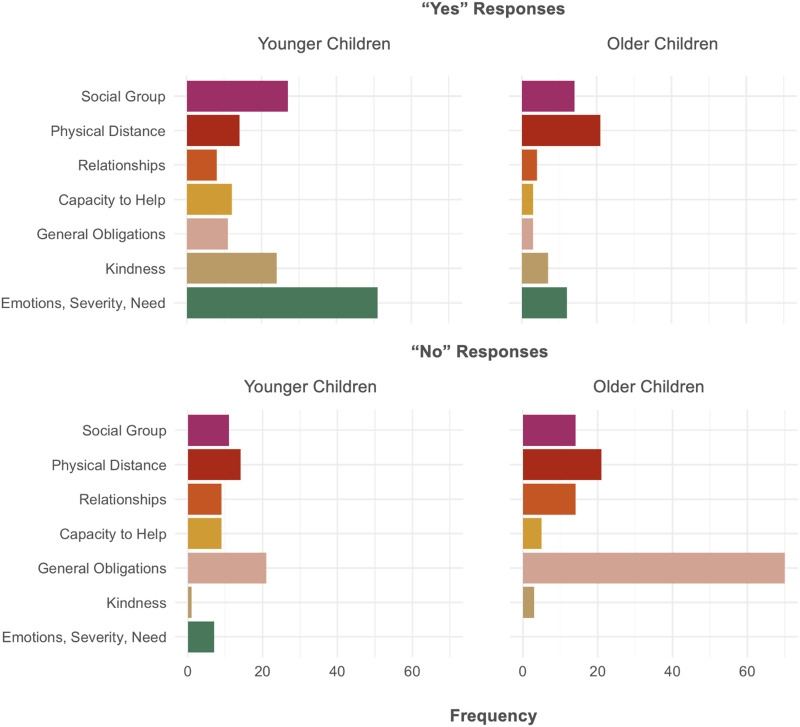
Frequencies of Participants’ Explanations.

Only a few participants indicated that they did not know (*n* = 11) and only a few responses were categorized as “other” (*n* = 14). To garner a better picture of the sorts of explanations children generated to justify helping or not, we examined the remaining categorizations of children’s responses depending on their Obligation Judgment (yes, no) and their Age Group (younger children, older children) (see Table 1 for aggregate frequencies). Younger children overwhelmingly justified an obligation to help by appealing to concerns related to urgency, such as emotions (e.g., “he looks sad”), severity (e.g., “she might have broken her leg”), and need (e.g., “he needs help”) (Figure 6). Older children, on the other hand, generated explanations in line with all seven categories. When justifying helping as non-obligatory, older children overwhelmingly referred to the notion that one is not obligated to do anything (e.g., “everybody has their own right to do everything and they’re free to say no”). The modal response in younger children also involved appealing to autonomy and the lack of individual obligation, though they provided explanations across all seven explanations in similar frequency generally.

### Discussion

Like Study 1, Study 2 revealed that younger children are much more inclined to describe individuals as obligated to help others compared to older children and adults. For Physical Distance, we found the same pattern of results within each age group as in Study 1; younger children did not consider physical distance while older children and adults did. The age-related comparisons were different from Study 1 though. Here, younger children were more sensitive to physical distance compared to older children but not adults, while older children were not more sensitive to physical distance compared to adults. Because of this difference in age effects between Study 1 and 2, we collapsed across both studies and found evidence for more consistent age-related effects where younger children were less differentiating than both older children and adults (as was the case in Study 1). This finding lends credibility to the notion that younger children are relatively less inclined to consider physical distance when ascribing obligations relative to older children and adults.

For Social Distance, we did not find the same pattern of results within each Age Group. In this study, younger children were sensitive to social distance and their degree of differentiation did not differ from older children or adults. The difference in younger children’s responses between Study 1 and 2 may be due to the stronger social group (rather than social distance) manipulation deployed in Study 2. In line with this, younger children’s responses in Study 1 were credibly different from Study 2. This difference is consistent with work that shows that ingroup biases tend to peak in early childhood and subsequently diminish, perhaps due to social desirability concerns (Rizzo et al., 2018; Rutland & Killen, 2015; Wilks et al., 2019; Yee & Brown, 1992). Here, both younger children and adults showed a tendency to consider social group membership when ascribing obligations, while children in middle childhood did not consider social group membership.

Finally, we examined children’s explanations for why others either do or do not have an obligation to help. Children who judged individuals as obligated to help (predominantly younger children) cited the severity of the situation, such as “really needing help.” Children who judged individuals as not obligated to help (predominantly older children) cited the belief that individuals do not have obligations to do anything. These data suggest that the extent to which distance, social relationships, and other factors shape our perceived obligation is also affected by a general belief about whether we have any obligations to help others.

## GENERAL DISCUSSION

Do you have less of an obligation to help a child who is suffering if that child happens to live on the other side of the world? This question has long been a topic of debate in philosophy and ethics (Igneski, 2001; Kamm, 2007; Singer, 1972; Timmerman, 2015; Unger, 1996). Despite the clear role of our psychology in shaping these judgments, there has been little work examining our intuitions about distance (e.g., Baron & Miller, 2000) and none exploring the developmental emergence of these intuitions. We conducted two studies examining how both physical and social distance shapes the perceived obligations of 5–9-year-old children and adults living in the United States.

In general, we found that younger children are far more likely to describe individuals as obligated to help others relative to both older children and adults, who tended to think that bystanders are generally not obligated to help others. This finding reflects past work showing that adults view helping as a supererogatory act, rather than an obligatory one, while children express a greater perceived obligation to helping others in general (e.g., Dahl et al., 2020).

For physical distance, the results were relatively straightforward: younger children were less sensitive to physical distance when ascribing obligations compared to older children and adults. This pattern was found in Study 1 and when collapsing across Study 1 and 2. In Study 2, we found this pattern when comparing younger children to older children but not when comparing younger children to adults. Given the results are better powered when collapsing across Study 1 and 2, we consider it reasonable to conclude that young children, relative to older children and adults, are less likely to factor physical distance into their obligation judgments. Put another way, young children think that we are obligated to help others regardless of how physically close or far away they are.

For social distance, adults judged individuals as more obligated to help when the individuals were described as behaving in similar ways (Study 1) or as members of the same group (Study 2). However, adults did not describe any of the potential helpers as obligated to help socially similar individuals or ingroup members, even though their obligation judgments did vary depending on social similarity and group membership (i.e., their scores were close to floor). That participants care about social distance aligns with previous work finding that both children and adults deeply care about groups and think others are more likely to act kindly toward ingroup members relative to outgroup ones (e.g., DeJesus et al., 2014; Everett et al., 2015; Jin & Baillargeon, 2017).

The age-related effects of social distance were more complex. In Study 1, we found that both younger and older children were less inclined to consider social distance compared to adults, but we did not find such an effect in Study 2. In Study 2, younger children considered social group membership, but older children did not (although neither group differed from one another). When comparing younger children’s responses between Study 1 and 2, they were credibly different from one another, suggesting that the more explicit group membership manipulation may have meaningfully changed the way younger children reason about social obligations. The particular pattern of results in Study 2 is consistent with work demonstrating that ingroup biases peak at around 4–5 years of age (Yee & Brown, 1992) and that older children are more likely than younger children to prioritize fairness over group concerns (Rizzo et al., 2018; Rutland & Killen, 2015; Wilks et al., 2019). That younger children consider social distance when individuals are described as members of the same group (Study 2), but not when they are described as merely similar (Study 1) or physically distant from one another (Study 1 and 2) aligns with past work demonstrating that children reason in nuanced and specific ways about explicitly defined social groups (e.g., Diesendruck & HaLevi, 2006; Jin & Baillargeon, 2017). Understanding what separates explicitly defined social groups from other kinds of social distance, such as mere similarity, is an important question for future research (see Jordan & Dunham, 2021).

We also examined participants’ reasoning for their choices. Participants who judged others as obligated to help (predominantly younger children) typically appealed to the severity of the situation. For example, young children described it as obligatory to help because someone is distressed or in harm’s way. These findings align with previous work revealing that adults (like children) are more inclined to describe strangers as obligated to help someone if the situation is urgent (Miller et al., 1990). Perhaps younger children’s inclination to judge others as obligated to help regardless of physical distance is, in part, a result of younger children perceiving the situations as more severe than older children and adults do.

By contrast, individuals who judged others as not obligated to help (predominantly older children) overwhelmingly justified this response by appealing to the broad idea that one is not obligated to do anything. Generally, this principle is consistent with autonomy and individualistic values in North American society (e.g., Wainryb, 2006). That is, more Western societies typically emphasize liberty and freedom, which may explain why older children shift toward appealing to such concerns when asked why the obligation to help does not extend to distant others. Consistent with this finding, past work shows that university students in India feel more obligated to help others, regardless of distance, than those in the United States (Baron & Miller, 2000). Further cross-cultural work could explore this possibility by examining how children in different societies reason about the obligation to help distant others.

Collectively, the present work raises a question regarding why younger children tend to cast a wider net of obligation (with the apparent exception of social group). One explanation for the present findings is that younger children reflexively indicate “yes” regardless of the situation. Against this possibility, other work finds that children are willing to respond “no” to similar obligation questions in different contexts. For example, younger children do not think that strangers are obligated to help if they are unaware of the person-in-need (Marshall et al., 2022), nor do they think that individuals have an obligation to act against their interests or in immoral ways (e.g., Miller et al., 1990). Another possible explanation is that younger children reason about the phrase “have to” differently than older children and adults do. Although this is possible, previous research has found that 4-year-olds distinguish between what an agent “has” to do, “likes” to do, or “usually” does (Kalish & Lawson, 2008). Further, other work using similar methods as our study finds that, although children’s obligation judgments (“have to”) tend to overlap with expectation judgments (“will do”), their expectation judgments do not explain their obligation judgments. This suggests that younger children do differentiate between obligation and expectation (Marshall et al., 2022).

It seems, then, that children perceive a wider net of obligation. There are several possible explanations for this. One appeals to social learning. It could be that younger children frequently hear narratives from their teachers, parents, and peers that helping is valuable under all circumstances and therefore espouse such notions in the present task (e.g., “You should help your friends” or “You should share your toys”). Older children may also hear these narratives but have greater exposure to more nuanced discussions about the nature of prosocial obligations. It is also possible that children’s conception of distance meaningfully differs from adults’ conceptions. Although younger children may understand that some people live far away in an abstract sense, they may have a less concrete understanding of this than adults, who have likely had a greater chance to travel and understand distance. This could lead to younger children placing less weight on physical distance, as identified here.

Young children’s tendency to conceptualize obligations more broadly relative to older children makes sense from an evolutionary perspective. To initially conceptualize obligations in an undifferentiated way is more cognitively efficient and also adaptive to the contexts in which children usually grow up. Children are usually surrounded by trusted others at young ages and only later come to interact with those who live far away or who are socially dissimilar. It may be cognitively easier for children to first learn a basic rule (helping is the right thing to do) and then later learn whom one should and should not help depending on social circumstances. Supporting this, other research has found that, at younger ages, children have more difficulty in weighing conflicting moral concerns and, as a result, default to assuming that helping is good regardless of the circumstances (Dahl et al., 2020; Marshall et al., 2022).

There are, of course, limits to what we can infer from these results. Some limitations pertain to our methodology. While we manipulated both social and physical distance and specified that the characters did not know each other, there are other potential confounds that come with physical distance. First, Nagel and Waldmann (2013) reveal that physical distance does not influence prosocial helping when it is isolated from other factors such as informational directness (i.e., how available the information about the victim’s need is), increased efficaciousness (i.e., likelihood of helping effectively), and shared group membership (i.e., being a part of the same cultural group). Although we in part accounted for efficaciousness and shared group membership, we did not directly account for informational directness. Given that children’s explanations differentially appealed to the seriousness of the victim’s need, future work could further investigate how children reason about obligations while better controlling for the seriousness of the situation.

Second, telling participants that it is *possible* to help both close and far individuals does not necessarily indicate that it is equally *easy* to help them. We attempted to remedy this concern by providing an example of how someone could easily help distant others by donating money and by noting in the question itself that the bystander could help the person-in-need. However, in reality it is often (but not always) easier to donate to individuals who are closer. For example, it can be more challenging to send money to an individual in another country relative to your own, but it is not harder to donate to an international charity relative to a domestic one. It is plausible adults are aware of these dynamics and that their obligation judgments about physically close and distant others are driven by such concerns. We think this is less relevant for older children, as they are less likely to be aware of the logistics associated with donating money but still felt fewer obligations to distant others. Nonetheless, future research on distance bias could better account for these factors by explicitly manipulating them and examining the extent to which describing helping as easier or harder influences obligation judgments.

Relatedly, the specific example used to explain how one could help someone far away (i.e., by donating money) may have played a role in children’s decreasing obligation judgments at older ages and adulthood. Specifically, older children and adults may have a more concrete understanding of the value of money and cost to the individual. In support of this, we know that children do consider cost when making moral judgments about helpfulness (Sierksma et al., 2014), and it is possible that older children perceived donating money as more costly than younger children.

There are also limitations associated with our Social Distance manipulation. It is possible that children were not conceptualizing individuals as particularly socially distant from one another. Although we describe individuals as different “types” (as defined by socially-relevant behaviors), participants may have still interpreted these individuals in the test trials as having different individual preferences for certain foods, music, and games rather than as acting in line with behaviors that define what “type of person” someone is. This distinction matters because children do not expect people within a group to hold similar preferences (Henderson & Woodward, 2012; Kalish, 2012; Weatherhead et al., 2016). In this way, children may have thought of these individuals as merely holding different preferences but as part of the same group (perhaps American citizens), limiting the extent to which the individuals are perceived as socially distant. Fortunately, Study 2 helps to remedy this concern because the individuals are explicitly labelled as members of groups, but it is still possible that children conceptualized these individuals as members of the same higher-order group because they were all engaging in at least familiar behaviors. Future work could elaborate on these ideas by differentiating between the groups in even further ways, such as describing the groups as adhering to different norms or conventions.

Finally, there are limitations of our sample. Almost all of our participants were White and all were recruited from the United States; a typical WEIRD culture (Henrich et al., 2010; Nielsen et al., 2017). Because of this, we do not know whether the present results would generalize to other groups within the United States or to other cultures across the world. Relatedly, the stimuli depicted White characters because previous research has found that children’s sense of obligation can differ as a function of race (Weller & Lagattuta, 2013); we did not want to introduce a confounding factor by presenting the targets as members of differing racial groups. Nonetheless, future work should consider both the racial and ethnic background of the participants and the stimuli to explore how these factors shape our perceived obligations. Given past work showing meaningful cultural differences in adults’ perceived obligation to distant others (e.g., Miller et al., 1990), we consider this a very important area of future research.

Across two studies with adults and children from 5–9 years of age, we find strong evidence that younger children consider strangers more obligated to help one another than older children or adults. We also find that distance generally reduces our perceived obligations towards others, but that this effect is much stronger for adults than children (and for older children than younger children), and also more reliable for physical distance than social distance. Tentatively, these results suggest that it may be erroneous to attribute moral beliefs about our (lack of) obligations to distant others to an innate or natural bias. Instead, we find that adults, compared to children, may be more likely to differentiate between the child drowning in a nearby pond and another dying of a preventable disease in a country far away.

## ACKNOWLEDGMENTS

This research was partly funded by the Klaus J. Jacobs Research Prize, awarded by the Jacobs Foundation. Thank you to Paul Bloom for his feedback on this manuscript and to Zachary Horne for his invaluable support with the statistical analyses. Thank you also to Maryam Khan and Amy Lu for their help with coding. Finally, thank you also to the children and families who participated in the experiments.

## AUTHOR CONTRIBUTIONS

Conceptualization: JM, MW; Data Curation: MW; Formal Analysis: JM; Methodology: JM, MW; Writing – Original Draft: JM, MW; Writing – Review & Editing: JM, MW.

## Notes

^1^ Images are copyrighted by and used by permission of VYOND™. VYOND is a trademark of GoAnimate, Inc., registered in Australia, Brazil, the European Union, Norway, the Philippines, Singapore, Switzerland, and the United Kingdom.^2^ Beyond these four trials, we also included four trials where we asked about expectation judgments (“If X needed help and Y could help her, do you think Y will help X? Yes or no?”). Importantly, our pre-registration specified that obligation judgments were the main dependent variable of interest given our research question, and the expectation judgments revealed similar developmental patterns as the obligation ones. For these reasons and also for brevity’s sake, we have placed all the expectation data in the Supplemental Online Materials (SOM).^3^ We examined these two models in part because our primary research questions were centered around age-related changes in reasoning about physical and social distance, although see the RMarkdown file for full model outputs of the Physical Distance x Social Distance interaction. In short, that interaction was not significant.^4^ Because these are Odds Ratios, ranges that overlap 1 are consistent with effects in both the positive and negative direction.^5^ This sample size is one child short of our plan because we determined after completing data collection that one child had already participated in Study 1 and therefore excluded them from the final sample.^6^ We again included four expectation trials that are reported in full in the SOM; the pattern of results found for expectation judgments do not differ from obligation judgments.

## Supplementary Material


