# Supplementary material for: Does Distance Matter? How Physical and Social Distance Shape Our Perceived Obligations to Others
Source: Open Mind (Camb). 2024 May 5;8:511–34. doi: 10.1162/opmi_a_00138 (PMC11093409; doi:10.1162/opmi_a_00138)

Supplemental Material for

**Does distance matter?**

**How physical and social distance shape our perceived obligations to others**

Table of Contents

[1 Study 1 2](#_Toc157074288)

[1.1 Obligation: Age Group Main Effect 2](#_Toc157074289)

[1.2 Obligation: Age (continuous) Analyses 3](#_Toc157074290)

[1.3 Expectation Analyses 5](#_Toc157074291)

[2 Study 2 8](#_Toc157074292)

[2.1 Obligation: Age Group Main Effect 8](#_Toc157074293)

[2.2 Obligation: Age (continuous) Analyses 9](#_Toc157074294)

[2.3 Expectation Analyses 11](#_Toc157074295)

[3 Comparing Study 1 and 2 14](#_Toc157074296)

[3.1 Visualization 14](#_Toc157074297)

# Study 1

## Obligation: Age Group Main Effect

**Supplementary Figure 1**

*Conditional Effects Plot of Age Group effect for Obligation Judgments*


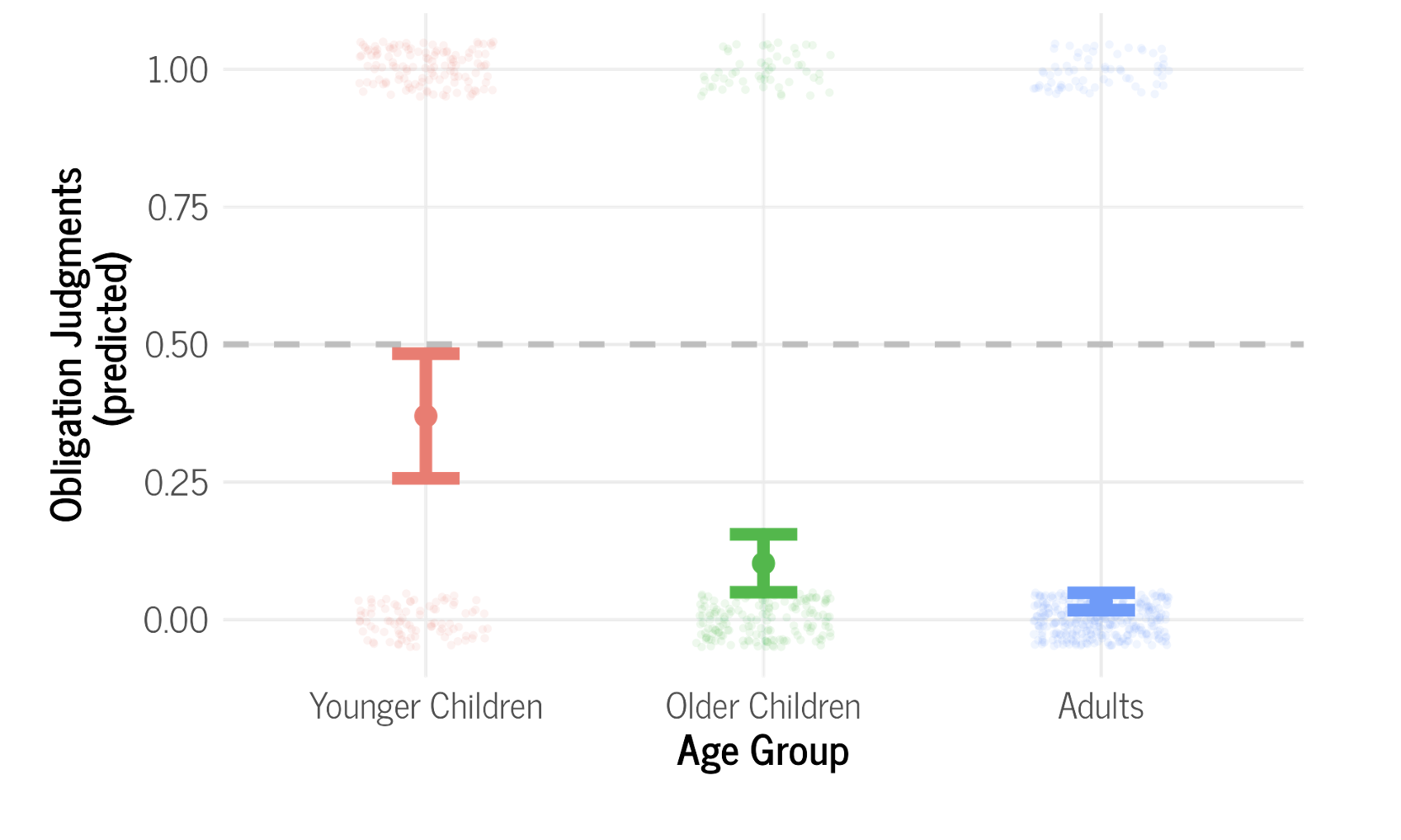


*Note*. The dot represents the mean of the posterior distribution of the estimate. Error bars are +/- 1 SE of the mean. The data points are jittered for readability.

## Obligation: Age (continuous) Analyses

As explained in the main manuscript, we initially pre-registered that we would examine children’s judgments only because, at the time, we had not collected adult data. Because we were ultimately interested in how children differed from adults, we divided children into two groups so that we could compare children to adults. That being said, we still wanted to examine just children, while considering age as a continuous predictor. When doing so, we fit two Bayesian logistic models: one included the interaction between Age (continuous) and Physical Distance (close, far) and the other included the interaction between Age (continuous) and Social Distance. These models mirror the main models found in the manuscript. For the model including the Age x Physical Distance interaction (Supplementary Figure 2A), we found that younger children were less differentiating on the basis of physical distance relative to older children, Odds Ratio = 0.74, 95% Credible Interval [0.58, 0.95]. For the model including the Age x Social Distance interaction (Supplementary Figure 2B), we did not find that younger children were less differentiating on the basis of social distance relative to older children, Odds Ratio = 0.95, 95% Credible Interval [0.67, 1.09]. In both cases, the results mirror the results in the main manuscript.

**Supplementary Figure 2**

*Conditional Effects Plots of (A) Physical Distance x Age and (B) Social Distance x Age*


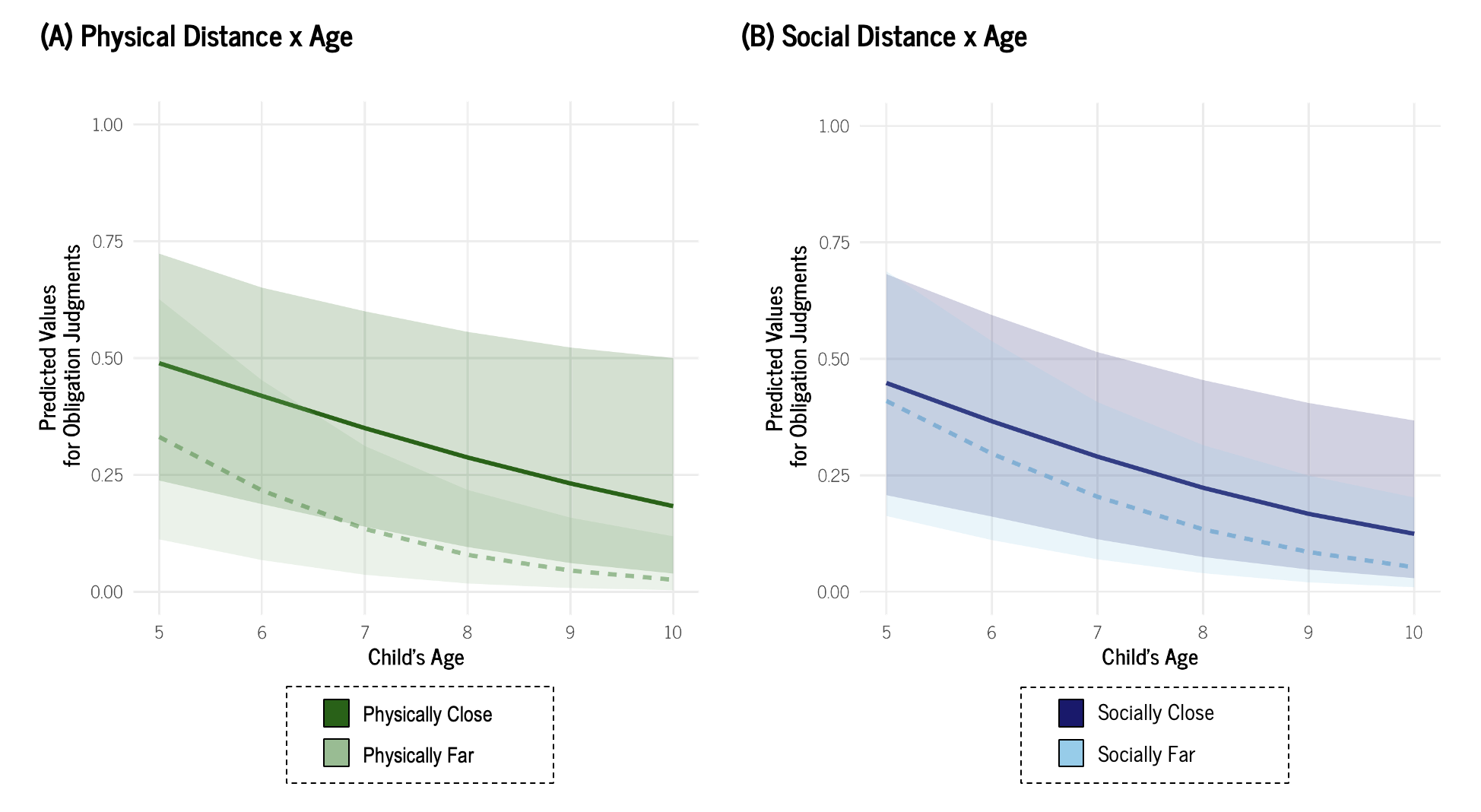


*Note*. Error bars are +/- 1 SE of the mean.

## Expectation Analyses

As mentioned in the main text, we also measured children’s expectations of helping behavior. Our primary research question centered around children’s obligation judgments, which is why those judgments are showcased in the manuscript. Nonetheless, better understanding whether children’s expectations of helpfulness are sensitive to both social and physical distance is of interest. We followed the same general structure in terms of the analyses. First, we fit a Bayesian logistic mixed effects model that included a three-way interaction between Physical Distance, Social Distance, and Age Group. As was the case with obligation judgments, the coefficients associated with a three-way interaction were not credibly different from zero. For this reason, we next looked at two models, one with the Physical Distance x Age Group interaction and another with Social Distance x Age Group interaction.

For the Physical Distance x Age Group interaction model, we also generally found the same results as was the case with obligation judgments. Unlike obligation judgments, younger children did differentiate between the likelihood that a bystander will help depending on physical distance, Odds Ratio = 0.42, 95% Credible Interval [0.22, 0.79]. Older children were more differentiating on the basis of physical distance compared to younger children, Odds Ratio = 0.22, 95% Credible Interval [0.08, 0.54]. Adults too were more differentiating than younger children, Odds Ratio = 0.39, 95% Credible Interval [0.18, 0.82]. Older children’s differentiation did not credibly differ from adults’, Odds Ratio = 1.79, 95% Credible Interval [0.72, 4.67]. The relevant estimates and standard errors can be found in the RMarkdown. Supplemental Figure 3A illustrates the Physical Distance x Age Group interaction.

For the Social Distance x Age Group interaction model, we also generally found the same results as was the case with obligation judgments. Unlike obligation judgments, younger children did differentiate between the likelihood that a bystander will help depending on social distance, Odds Ratio = 0.52, 95% Credible Interval [0.23, 0.96]. As was the case for obligation judgments, older children were not more differentiating on the basis of social distance compared to younger children, Odds Ratio = 1.25, 95% Credible Interval [0.54, 2.86]. Adults, though, were more differentiating on the basis of social distance compared to younger children, Odds Ratio = 0.24, 95% Credible Interval [0.11, 0.50]. Interestingly, older children were more differentiating on the basis of social distance compared to adults, Odds Ratio = 0.19, 95% Credible Interval [0.08, 0.44]. Supplemental Figure 3B illustrates the Social Distance x Age Group interaction.

**Supplemental Figure 3**

*Conditional Effects Plots of (A) Physical Distance x Age Group and (B) Social Distance x Age Group*


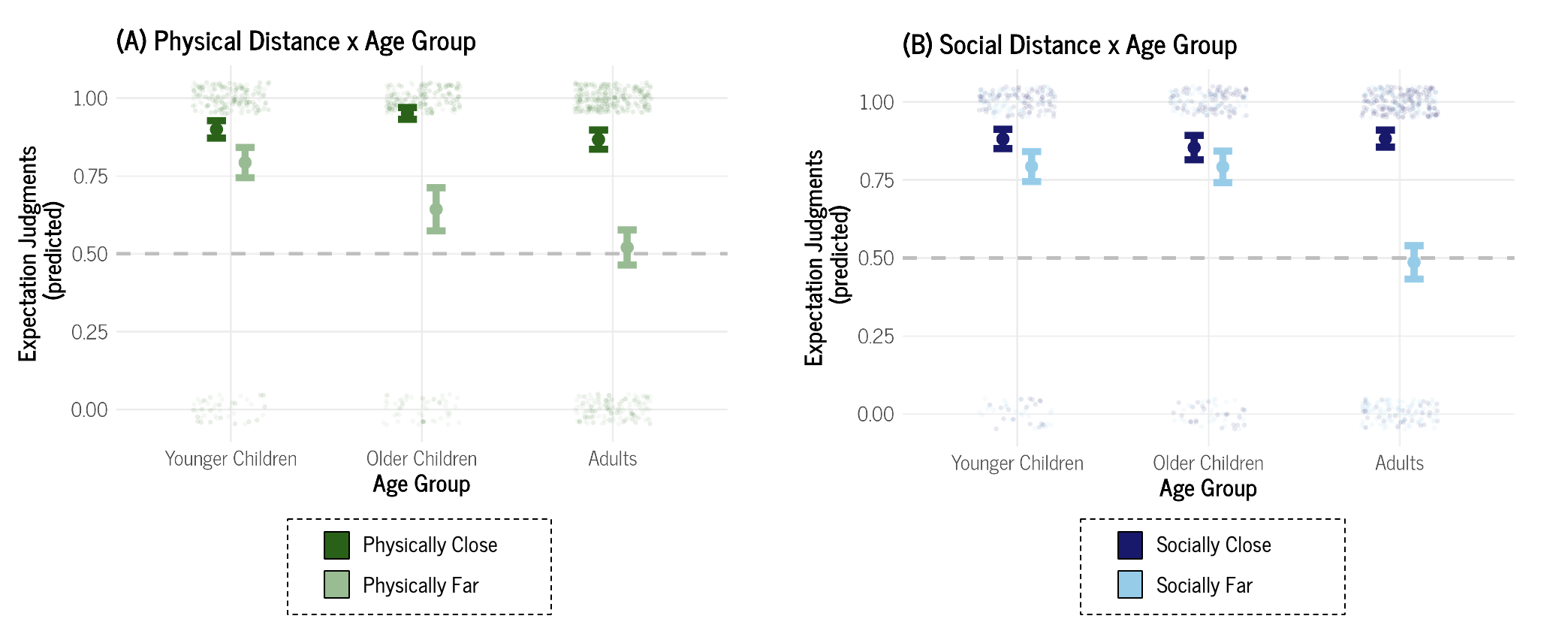


*Note*. The dot represents the mean of the posterior distribution of the estimate. Error bars are +/- 1 SE of the mean. The data points are jittered for readability.

As was the case for obligation judgments, we wanted to generally examine whether younger children held higher expectations about the likelihood of helping others compared to older children and adults. To do so, we fit a Bayesian logistic mixed effects model with a main effect of Age Group. As illustrated by Supplemental Figure 4, all age groups thought the bystander would help. Younger children and older children did not differ in their predictions about helping behavior, Odds Ratio = 0.88, 95% Credible Interval [0.48, 1.61], although younger children did differ from adults, Odds Ratio = 0.48, 95% Credible Interval [0.28, 0.81]. Older children as well differed from adults, Odd Ratio = 0.55, 95% Credible Interval [-1.16, -0.07].

**Supplemental Figure 4**

*Conditional Effects Plot of Age Group effect for Expectation Judgments*


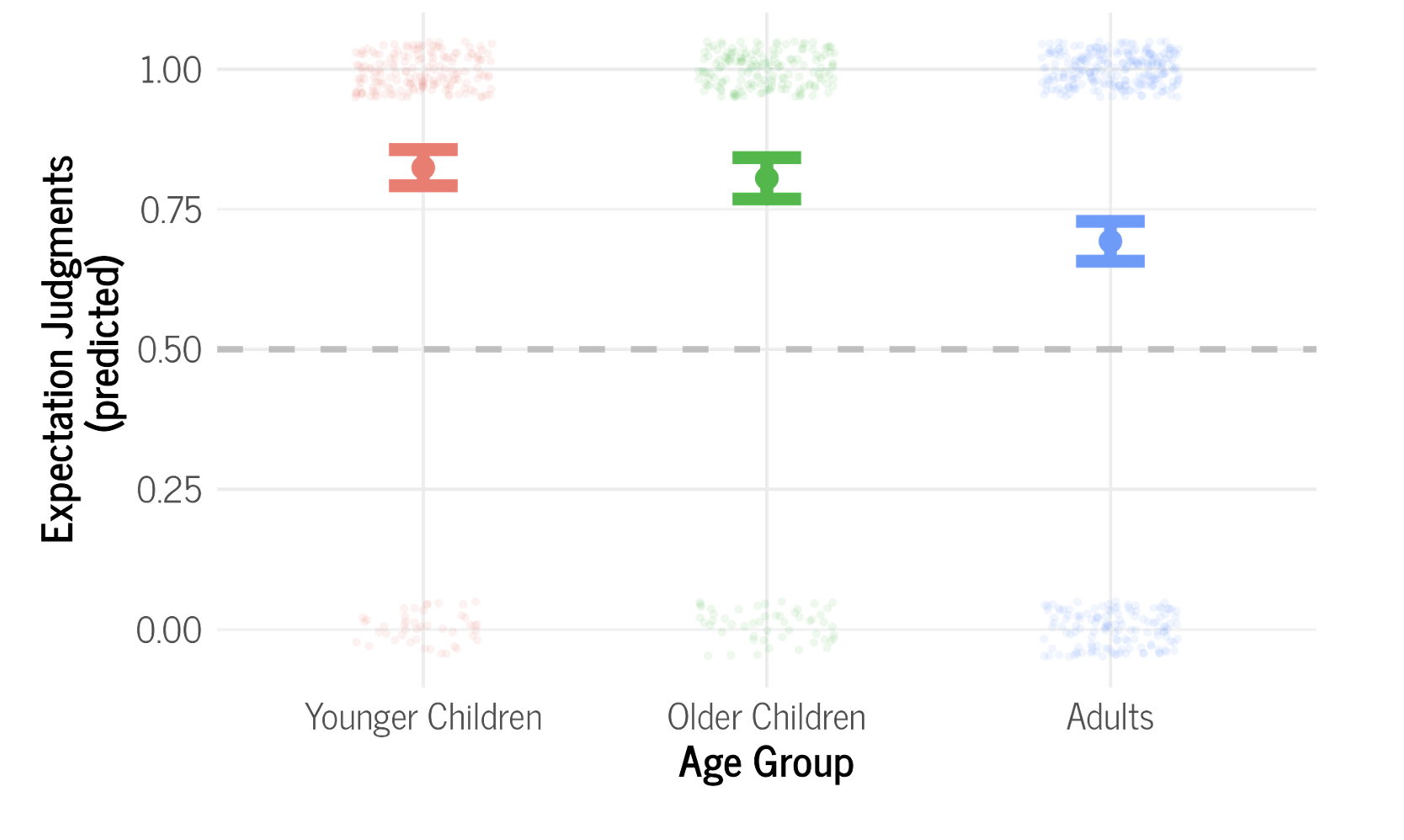


*Note*. The dot represents the mean of the posterior distribution of the estimate. Error bars are +/- 1 SE of the mean. The data points are jittered for readability.

# Study 2

## Obligation: Age Group Main Effect

**Supplementary Figure 5**

*Conditional Effects Plot of Age Group effect for Obligation Judgments*


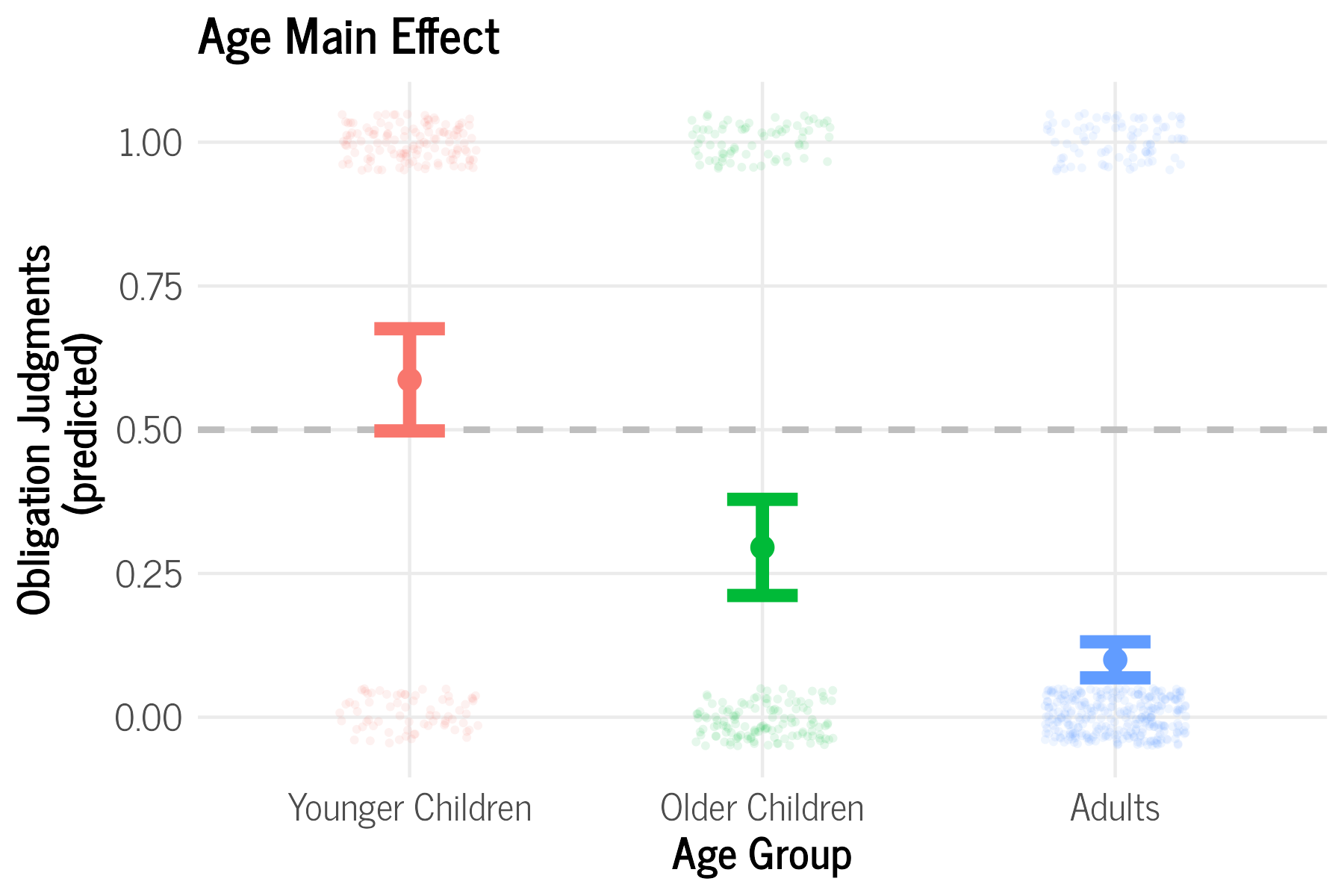


*Note*. The dot represents the mean of the posterior distribution of the estimate. Error bars are +/- 1 SE of the mean. The data points are jittered for readability.

## Obligation: Age (continuous) Analyses

For reasons described above under “Study 1,” we fit two Bayesian logistic models: one included the interaction between Age (continuous) and Physical Distance (close, far) and the other included the interaction between Age (continuous) and Social Group Membership. These models mirror the main models found in the manuscript. For the model including the Age x Physical Distance interaction (Supplementary Figure 6A), we found that younger children were less differentiating on the basis of physical distance relative to older children, Odds Ratio = 0.70, 95% Credible Interval [0.56, 0.87]. For the model including the Age x Social Group Membership interaction (Supplementary 6B), we did not find that younger children were less differentiating on the basis of social distance relative to older children, Odds Ratio = 0.87, 95% Credible Interval [0.70, 1.08]. In both cases, the results mirror the results in the main manuscript.

**Supplementary Figure 6**

*Conditional Effects Plots of (A) Physical Distance x Age and (B) Social Group Membership x Age*


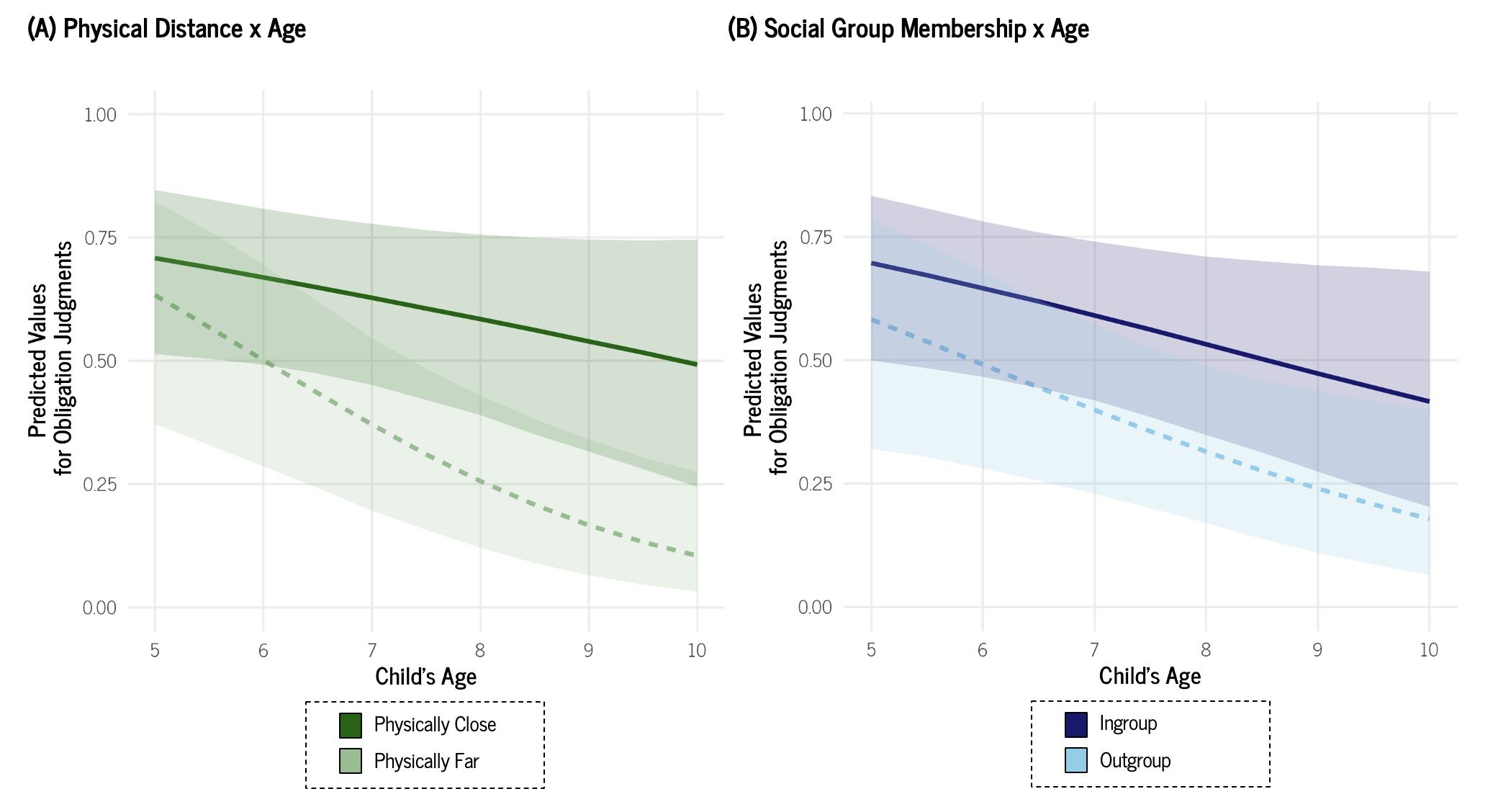


*Note*. Error bars are +/- 1 SE of the mean.

## Expectation Analyses

First, we fit a Bayesian logistic mixed effects model that included a three-way interaction between Physical Distance, Social Distance, and Age Group. As was the case with obligation judgments, the coefficients associated with a three-way interaction were not credibly different from zero. For this reason, we next looked at two models, one with the Physical Distance x Age Group interaction and another with Social Distance x Age Group interaction.

For the Physical Distance x Age Group interaction model, we found that younger children did differentiate between the likelihood that a bystander will help depending on physical distance, Odds Ratio = 0.42, 95% Credible Interval [0.22, 0.79]. Older children were more differentiating on the basis of physical distance compared to younger children, Odds Ratio = 0.22, 95% Credible Interval [0.08, 0.54]. Adults too were more differentiating than younger children, Odds Ratio = 0.39, 95% Credible Interval [0.18, 0.82]. Older children’s differentiation did not credibly differ from adults’, Odds Ratio = 0.61, 95% Credible Interval [0.28, 1.42]. The relevant estimates and standard errors can be found in the RMarkdown. Supplemental Figure 7A illustrates the Physical Distance x Age Group interaction.

For the Social Group Membership x Age Group interaction model, we found that younger children did differentiate between the likelihood that a bystander will help depending on social group membership, Odds Ratio = 0.40, 95% Credible Interval [0.21, 0.75]. Older children were not more differentiating on the basis of social distance compared to younger children, Odds Ratio = 0.56, 95% Credible Interval [0.23, 1.28]. Adults, though, were more differentiating on the basis of social group membership compared to younger children, Odds Ratio = 0.37, 95% Credible Interval [0.17, 0.81]. Older children’s sensitivity to social group membership did not differ from adults’, Odds Ratio = 0.67, 95% Credible Interval [0.29, 1.55]. Supplemental Figure 7B illustrates the Social Group Membership x Age Group interaction.

**Supplemental Figure 7**

*Conditional Effects Plots of (A) Physical Distance x Age Group and (B) Social Distance x Age Group*


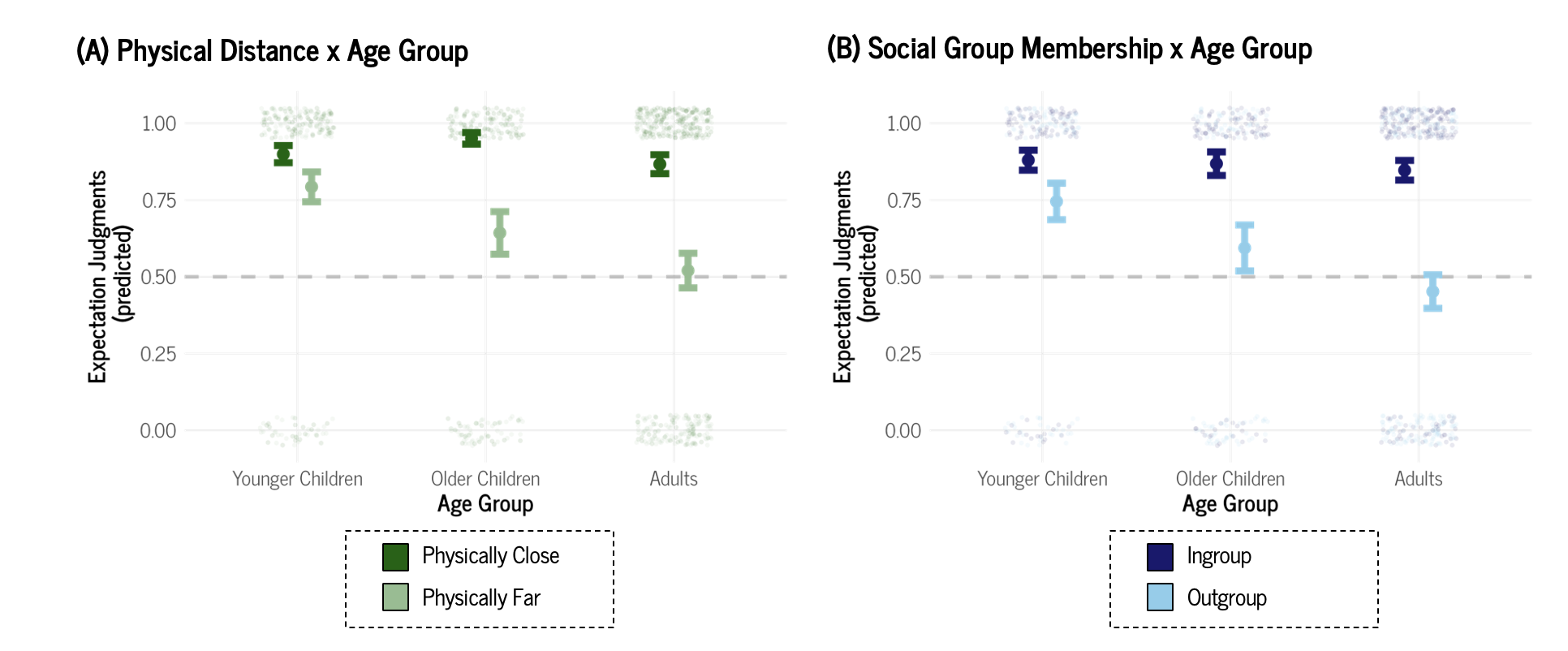


*Note*. The dot represents the mean of the posterior distribution of the estimate. Error bars are +/- 1 SE of the mean. The data points are jittered for readability.

As was the case for obligation judgments, we wanted to generally examine whether younger children held higher expectations about the likelihood of helping others compared to older children and adults. To do so, we fit a Bayesian logistic mixed effects model with a main effect of Age Group. As illustrated by Supplemental Figure 8, all age groups thought the bystander would help. Younger children and older children did not differ in their predictions about helping behavior, Odds Ratio = 0.68, 95% Credible Interval [0.36, 1.26], although younger children did differ from adults, Odds Ratio = 0.48, 95% Credible Interval [0.28, 0.81]. Older children did not differ from adults, Odd Ratio = 0.71, 95% Credible Interval [0.40, 1.22].

**Supplemental Figure 8**

*Conditional Effects Plot of Age Group effect for Expectation Judgments*


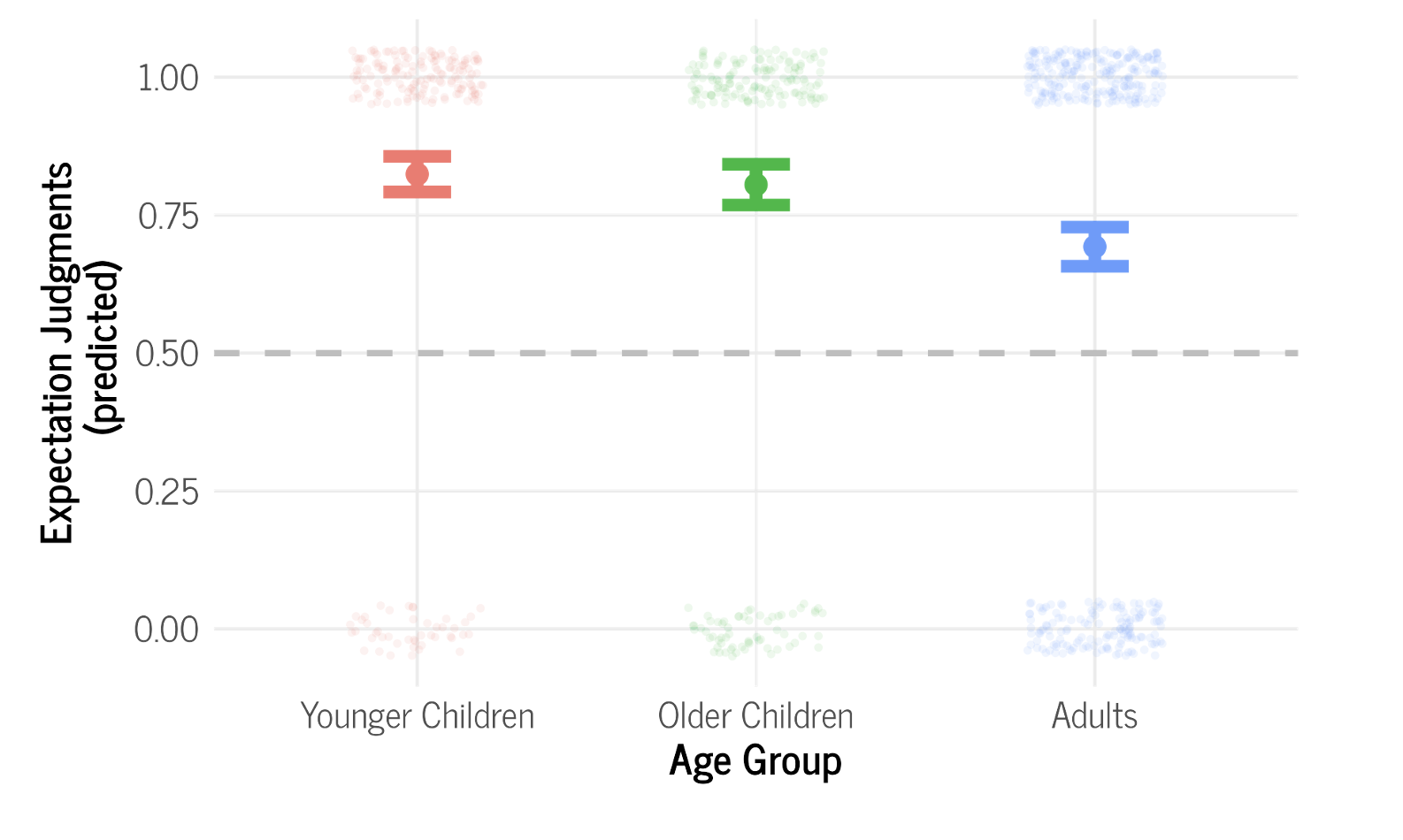


*Note*. The dot represents the mean of the posterior distribution of the estimate. Error bars are +/- 1 SE of the mean. The data points are jittered for readability.

# Comparing Study 1 and 2

## Visualization

**Supplemental Figure 9**

*Conditional Effects Plot of Social Distance x Study for Younger Children*


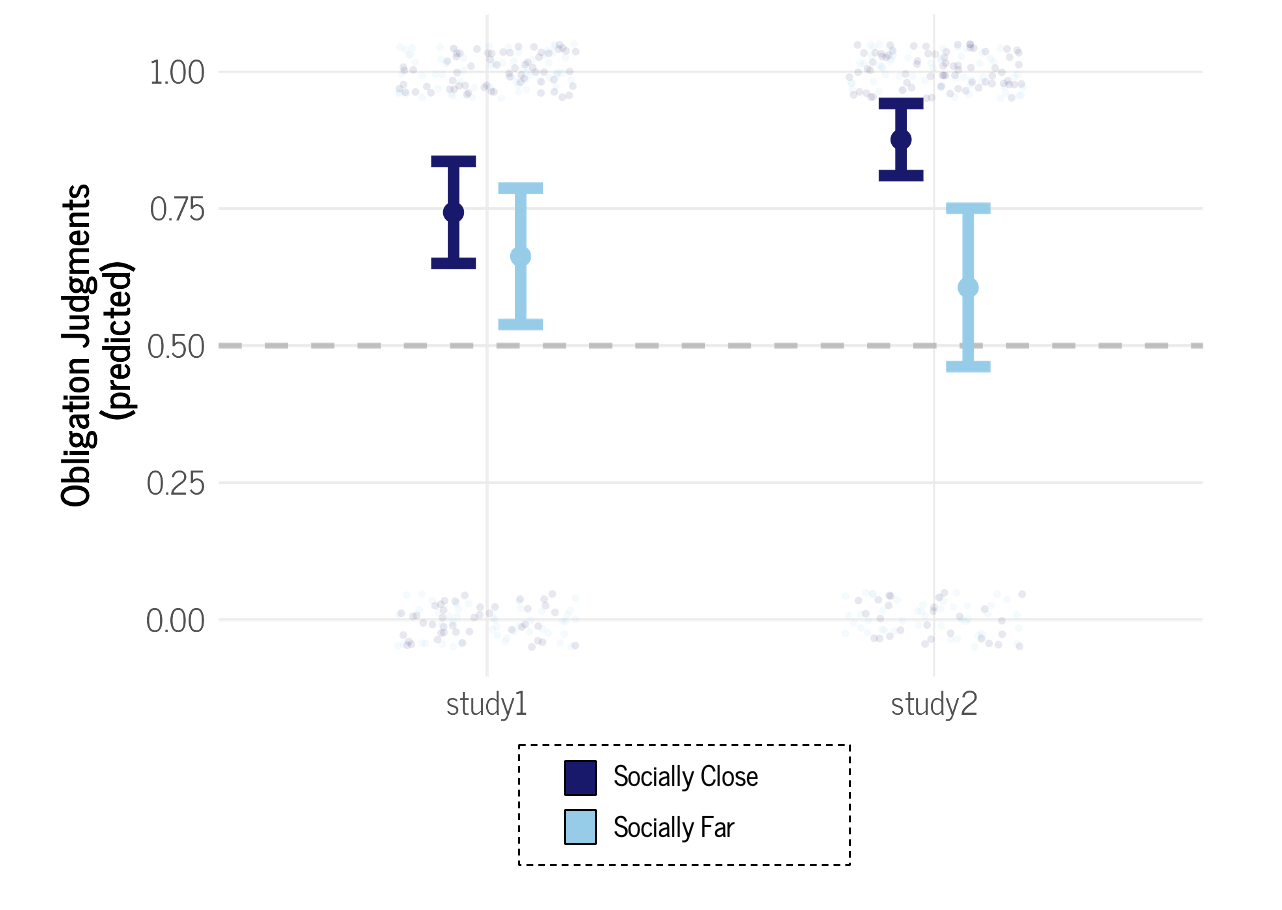

Supplement: Supplementary file 1 [file opmi-08-511-s001.docx]
